# Biophysical attributes that affect CaMKII activation deduced with a novel spatial stochastic simulation approach

**DOI:** 10.1371/journal.pcbi.1005946

**Published:** 2018-02-05

**Authors:** Ximing Li, William R. Holmes

**Affiliations:** Department of Biological Sciences, Neuroscience Program, Ohio University, Athens, Ohio, United States of America; Instytut Biologii Doswiadczalnej im M Nenckiego Polskiej Akademii Nauk, POLAND

## Abstract

Calcium/calmodulin-dependent protein kinase II (CaMKII) holoenzymes play a critical role in decoding Ca^2+^ signals in neurons. Understanding how this occurs has been the focus of numerous studies including many that use models. However, CaMKII is notoriously difficult to simulate in detail because of its multi-subunit nature, which causes a combinatorial explosion in the number of species that must be modeled. To study the Ca^2+^-calmodulin-CaMKII reaction network with detailed kinetics while including the effect of diffusion, we have customized an existing stochastic particle-based simulator, Smoldyn, to manage the problem of combinatorial explosion. With this new method, spatial and temporal aspects of the signaling network can be studied without compromising biochemical details. We used this new method to examine how calmodulin molecules, both partially loaded and fully loaded with Ca^2+^, choose pathways to interact with and activate CaMKII under various Ca^2+^ input conditions. We found that the dependence of CaMKII phosphorylation on Ca^2+^ signal frequency is intrinsic to the network kinetics and the activation pattern can be modulated by the relative amount of Ca^2+^ to calmodulin and by the rate of Ca^2+^ diffusion. Depending on whether Ca^2+^ influx is saturating or not, calmodulin molecules could choose different routes within the network to activate CaMKII subunits, resulting in different frequency dependence patterns. In addition, the size of the holoenzyme produces a subtle effect on CaMKII activation. The more extended the subunits are organized, the easier for calmodulin molecules to access and activate the subunits. The findings suggest that particular intracellular environmental factors such as crowding and calmodulin availability can play an important role in decoding Ca^2+^ signals and can give rise to distinct CaMKII activation patterns in dendritic spines, Ca^2+^ channel nanodomains and cytoplasm.

## Author summary

Ca^2+^ signals are commonly used by cells for various types of activities. In neurons, Ca^2+^ can regulate gene expression and dendritic spine enlargement, strengthen synaptic connectivity, and promote neural growth or even death. One important Ca^2+^ binding protein is calmodulin, which has a wide range of downstream effectors that relay and interpret Ca^2+^ signals. These downstream proteins have different calmodulin binding kinetics and can interact with calmodulin differently depending on Ca^2+^ signaling patterns and in particular, on signaling frequencies. In this study, we focus on one particular pathway that is thought to be important for learning and memory and involves calmodulin and the effector protein, Ca^2+^/calmodulin-dependent protein kinase II (CaMKII). We have developed a novel computational method that avoids the problem of combinatorial explosion inherent with modeling this pathway, and the method allows us to study the pathway in more detail than conventional methods. Experiments have shown that CaMKII activity is sensitive to Ca^2+^ signal frequency and our models demonstrate how this frequency dependence relies on the amount of Ca^2+^ input, calmodulin availability and the Ca^2+^ diffusion rate.

## Introduction

**Fig 1:**
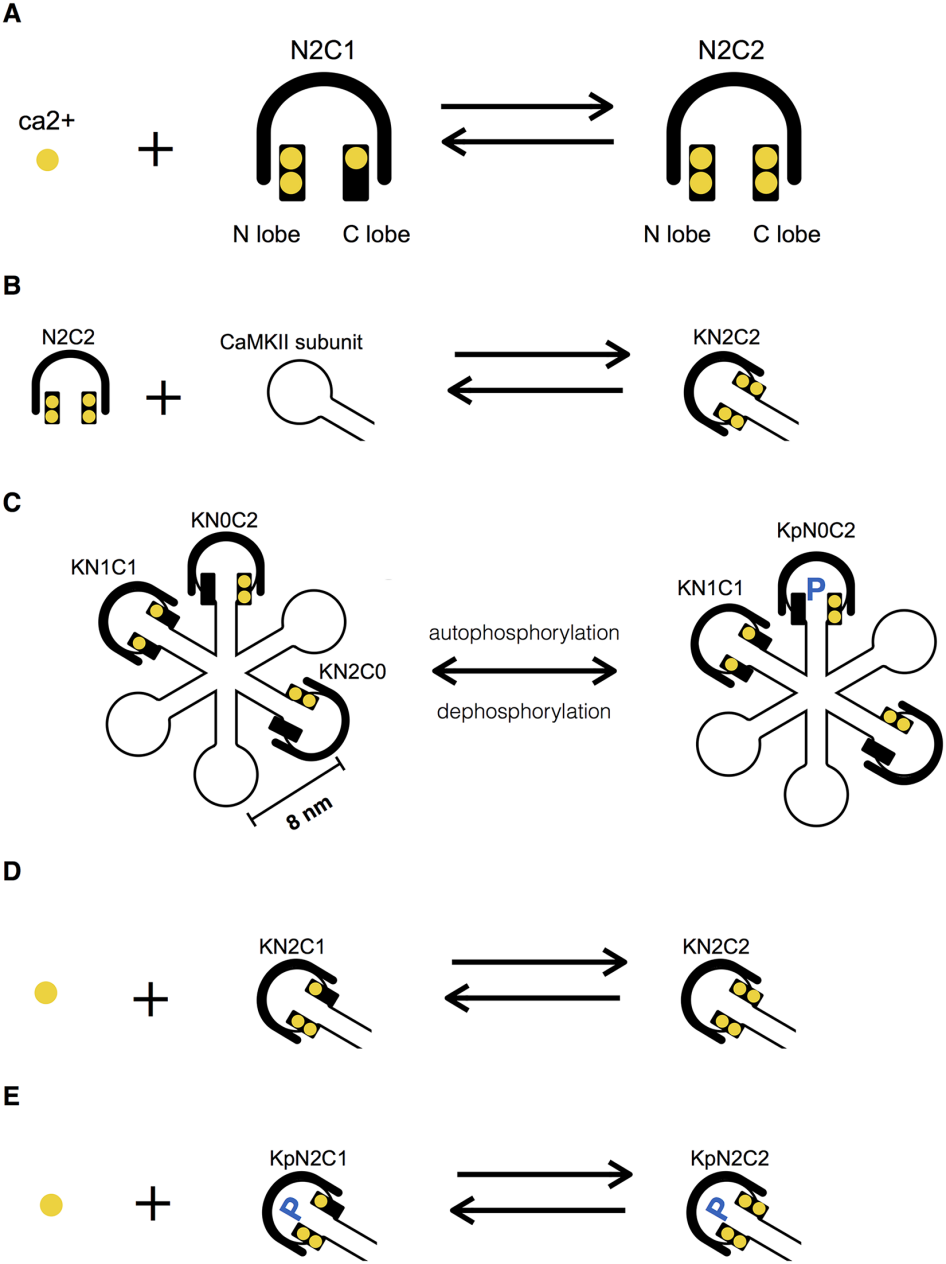
Types of Ca^2+^-CaM-CaMKII interactions modeled. (A) Ca^2+^ binds to a CaM molecule that already has 3 Ca^2+^ bound, making the CaM fully loaded. Each CaM has two lobes, an N lobe and a C lobe. Each lobe has 2 Ca^2+^ binding sites. (B) A CaM molecule binds to a CaMKII subunit, activating the subunit. Each subunit of the CaMKII holoenzyme can interact with CaM independently. (C) CaMKII autophosphorylation is neighbor sensitive and follows one direction (arbitrarily chosen as left to right here). When both a subunit and its neighbor to the left are activated, the subunit can be phosphorylated. (D) CaM bound to a CaMKII subunit interacts with Ca^2+^ at an increased affinity. (E) CaM bound to a phosphorylated CaMKII subunit also interacts with Ca^2+^ at a further increased affinity. Notation: K represents an unphosphorylated CaMKII subunit; Kp is a phosphorylated subunit; NiCj is CaM with i Ca^2+^ ions bound at the N lobe and j Ca^2+^ ions bound at the C lobe.

Calcium/calmodulin-dependent protein kinase II (CaMKII) is an important enzyme widely distributed in the central nervous system and other tissues including cardiac muscle [1–3]. In intracellular Ca^2+^ signaling, the Ca^2+^ sensor protein calmodulin (CaM) binds to and activates CaMKII [4–6]. Importantly CaM binding to CaMKII produces an allosteric change in CaM that increases its binding affinities for Ca^2+^ [7] (Fig 1). In neurons, CaMKII molecules play an essential role in long-term potentiation (LTP), our best model for the molecular basis of learning and memory [8]. In spines, activated CaMKII molecules interact with postsynaptic density proteins, facilitating actins to reorganize, leading to spine enlargement and upregulation of *α*-amino-3-hydroxy-5-methyl-4-isoxazolepropionic acid (AMPA) receptor numbers [4]. CaMKII molecules can also phosphorylate AMPA receptors to regulate channel conductance [4, 6] or form complexes with NMDA receptors [5]. In addition to synaptic functions, recent studies suggest that CaMKII in the soma can act as carriers to shuttle Ca^2+^-CaM into the nucleus [9, 10]. Ca^2+^-CaM molecules are then unloaded in the nucleus, and participate in the calcium/calmodulin dependent protein kinase IV (CaMKIV) cascade to activate nuclear transcription factors. Therefore, CaMKII molecules play a key role in excitation-transcription coupling.

The CaMKII molecule is a holoenzyme that consists of 12 subunits grouped in 2 rings [2, 5, 6, 11]. Each subunit contains an association domain allowing the formation of multimers, a regulatory domain with a CaM binding site and phosphorylation sites, and a catalytic domain to act as a kinase [2, 5, 6, 11]. Activities such as CaM binding and auto-phosphorylation result in various subunit states. For example, in the presence of Ca^2+^-CaM complexes, each subunit can bind a CaM molecule and expose its catalytic domain to phosphorylate the direct neighbor subunit at its Thr286 site (for *α*CaMKII) [12]. Once phosphorylated, the CaM unbinding rate decreases dramatically, leading to a prolonged activation of the subunit; we say, the CaM molecule is “trapped” by the CaMKII subunit [13]. Even when the CaM molecule unbinds, the CaMKII subunit stays activated, entering an autonomous state. Finally, Thr305/Thr306 can also be phosphorylated. There the threonine sites overlap with the CaM binding site and their phosphorylation blocks the binding of CaM molecules. In this case, the subunit becomes “capped”.

Researchers have long been interested in modeling and simulating CaMKII [14–23]. However, the structural complexity and multi-state nature of CaMKII present a technical challenge. A major problem is combinatorial explosion [24]. One CaMKII subunit has only a moderate number of states to consider, but with 6 or 12 subunits on a holoenzyme, the number of combinations of states for a holoenzyme becomes extremely large. This is a common problem in systems biology since large proteins usually consist of multiple subunits. Software that adopts a rule-based approach such as BioNetGen [25] can be used to expand the network based on a set of given reaction rules. Nevertheless, for a CaMKII holoenzyme, using BioNetGen to generate the network is still computationally intensive. After expansion with BioNetGen, a 6-subunit holoenzyme with four states for each subunit has 700 unique species and 12,192 unique reactions (See S2 File for detailed calculations).

The size of the network and the problem of combinatorial explosion grow substantially when more detailed kinetics are considered. For example, each CaM has 2 lobes and in total 4 Ca^2+^-binding sites. This can introduce 5, 9, 16 or more binding states depending on assumptions made [7, 19–21, 23, 26, 27]. Here we follow the experimental work of Linse et al. [28] and assume that each lobe binds Ca^2+^ independently and within each lobe, Ca^2+^ binding is cooperative. This gives rise to 9 distinct binding states. In addition, it is known that Ca^2+^ binding kinetics to CaM is different whether CaM is free, bound to CaMKII or bound to phosphorylated CaMKII [29–32]. Therefore, we allow each CaM state to have distinct kinetics to interact with a CaMKII subunit. Also, each CaMKII subunit exhibits a distinct phosphorylation rate depending on the state of the bound CaM [21, 29]. Consequentially, each subunit would potentially have 20 states. For simplicity, consider a 6-subunit CaMKII holoenzyme. Then for the holoenzyme, the total number of unique species alone reaches 10,668,140 (computed with the necklace function [33] see S2 File). It would be extremely time-consuming and not practical for BioNetGen to generate this network. To overcome this problem, most previous modeling studies of CaMKII have simplified the Ca^2+^-CaM-CaMKII reaction network by either modeling CaMKII as monomers [18, 21] or allowing only CaM fully-loaded with Ca^2+^ to interact with CaMKII subunits [14, 16, 17, 22]. However, Pepke et al. [21] has shown that a reaction network without sufficient kinetic resolution has limited predictive power for intracellular signaling events.

The intracellular environment presents another complication. Conventionally, biochemical reactions are modeled in a deterministic manner using Ordinary Differential Equations (ODEs). The underlying assumptions are that reactions occur in a spatially homogenous environment, reactants are abundant and not subject to stochasticity, and molecules diffuse sufficiently fast. The majority of previous modeling studies belong to this category except for a few that are stochastic or hybrid models [15, 18, 20, 27]. However, intracellular space is highly heterogeneous and compartmentalized [34, 35]. Reactions are often restricted to a small space. Structures such as the cytoskeleton, scaffold proteins or endoplasmic reticulum often act as diffusion barriers to slow down molecules. In addition, most previous modeling studies focus on the dendritic spine, where NMDA receptor-channels are the main Ca^2+^ providers.

In the present work, we focus on the soma where voltage-dependent Ca^2+^ channels (Ca_V_) provide the Ca^2+^ influx. Specifically, we examined Ca^2+^-CaM-CaMKII network activity near L-type Ca^2+^ channels, which constitute the major Ca^2+^ source in the soma. This is an area less studied in previous models, but plays a critical role in excitation-transcription coupling [10, 36]. To study this Ca^2+^-CaM-CaMKII signaling network and deal with the problem of combinatorial explosion, we modified the published and freely available simulator Smoldyn [37, 38]. Smoldyn is a particle-based stochastic simulator and has been used for simulating reaction and diffusion processes in cells. It works well for relatively simple reaction networks but not for holoenzymes such as CaMKII. We added new data structures to Smoldyn to describe the reaction network in a compact way. CaMKII holoenzymes are modeled as a collection of subunits. Each subunit has a set of binding sites. Subunits react independently and diffuse collectively. Reactions are defined between binding sites. The reaction network is stored in a hash table to allow for lookup during simulations when reactants collide. Therefore, expanding and loading a complete network is not required. We tested and verified these modifications and then created a detailed Ca^2+^-CaM-CaMKII network to examine factors that affect the frequency dependence of CaMKII activation. We found that the total Ca^2+^ influx amount, as well as Ca^2+^ diffusion rate and CaM availability, can change the dependence of CaMKII phosphorylation on Ca^2+^ input frequencies. Meanwhile, driven by Ca^2+^ input with a given frequency, CaM species travel through an altered pathway along with the change of CaMKII phosphorylation pattern.

## Results

### Testing of the simulator modifications

**Fig 2:**
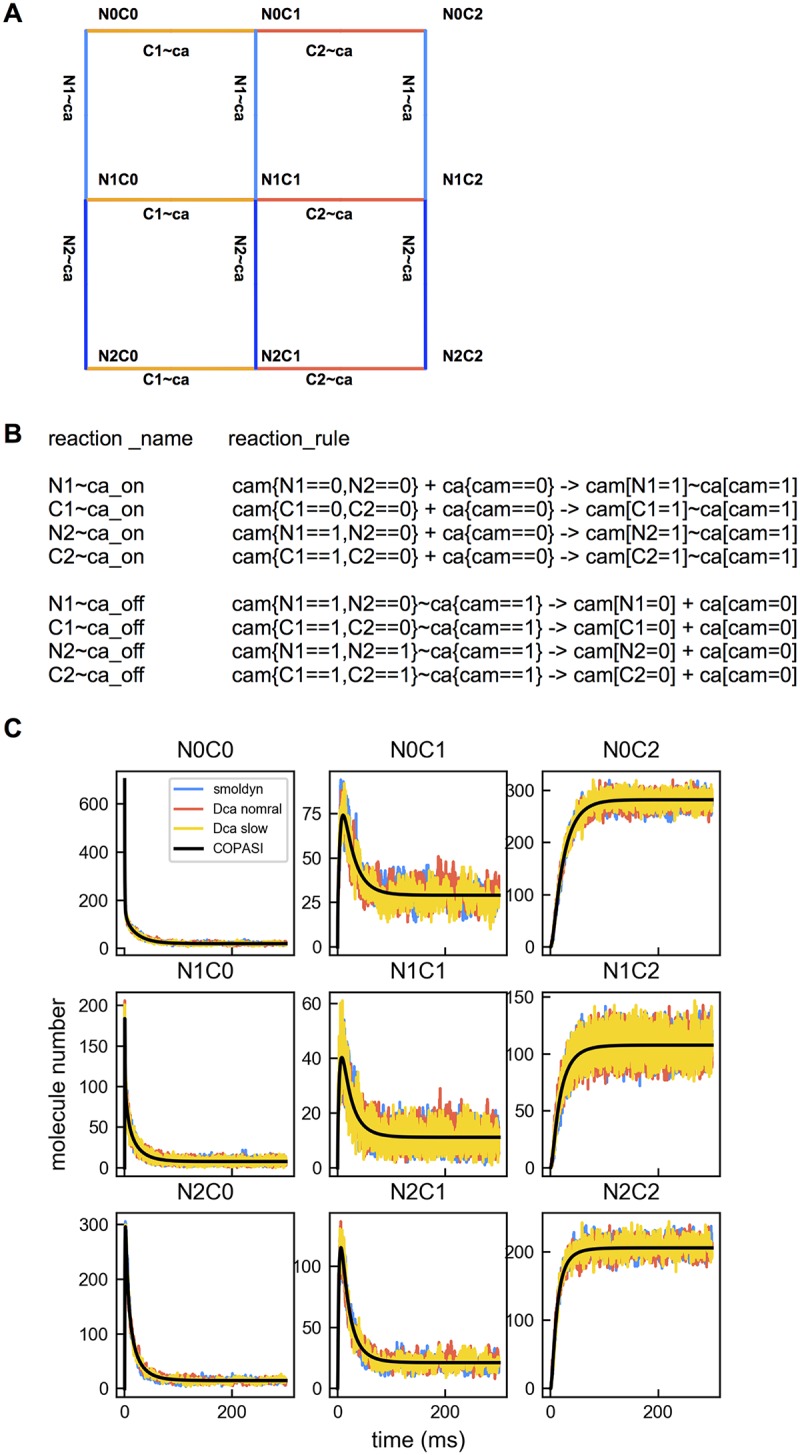
Ca^2+^-CaM interaction network. (A) Ca^2+^ and CaM interactions can produce 9 different states of CaM. CaM has two lobes, an N lobe and a C lobe, each of which has 2 Ca^2+^ binding sites. Binding sites on the lobes are designated N1, N2, C1 and C2. The N lobe and C lobe bind Ca^2+^ independently of each other, but Ca^2+^ binding within a lobe is cooperative. Thus, site N2 cannot bind Ca^2+^ unless N1 has already bound Ca^2+^ and a similar rule applies to sites C2 and C1. In the figure N0C0 represents apoCaM (no Ca^2+^ bound), “N2” represents binding sites N1 and N2 both bound with Ca^2+^ and “C2” represents similarly that both C1 and C2 have Ca^2+^ bound, “N1” and “C1” represent only the “N1” site and only the “C1” site have Ca^2+^ bound. Edges are labeled to indicate bidirectional interactions, including both bindings and unbindings. For example, “N1∼ca” means interactions between a Ca^2+^ ion and a CaM at the N1 site. Likewise, “C1∼ca” means interactions between a Ca^2+^ ion and a CaM at the C1 site. (B) The same reactions between Ca^2+^ and CaM as shown in A are represented using the syntax of the modified simulator. The binding and unbinding rate constants are labeled on the left. “+” represents a binding reaction; “∼” represents molecules that are bound. “{}” on the left-hand side of the reaction specifies the states of the reactant binding sites or the conditions for a reaction to occur. On the right-hand side, binding sites involved in the reaction are assigned new values. Notice the “==” sign is used on the left-hand side, but the “=” sign is used on the right-hand side. The “==” represents True (==1) or False (==0), whereas the “=” denotes an assigning operation. (C) The time course of each CaM state simulated using the original Smoldyn (blue), modified Smoldyn at default Ca^2+^ diffusion (red), half Ca^2+^ diffusion (yellow line) and COPASI (black lines).

We first tested the modifications to Smoldyn used in this paper (see Methods). The reaction and diffusion kinetics of Smoldyn have been thoroughly validated in the past [37], but we still needed to validate that our modifications for molecular complex management were working properly. Our first test used a Ca^2+^-CaM network (Fig 2) (Model 1). The 4 Ca^2+^ binding sites on CaM give rise to a total of 9 different binding states of a CaM molecule. For the reaction volume, we use a 500 nm × 500 nm × 500 nm cube with all sides being reflective. The size of this volume is what might be used to study a Ca^2+^ channel nanodomain (the region up to 100nm from the channel pore) or a small dendritic spine head. Initially, the cube contains 3000 Ca^2+^ ions and 700 apoCaM molecules, equivalent to 39.867 μm and 9.302 μm respectively. The concentrations are chosen to produce an observable amount of 4-Ca^2+^ bound CaM at the steady state. All molecules are initially uniformly distributed. We tested reactions using two different Ca^2+^ diffusion constants, 2.2 × 10^−6^ cm^2^ s^−1^ [39, 40] and half of this 1.1 × 10^−6^ cm^2^ s^−1^. A detailed description of the model and other testing models is in S1 File and S1 Table.

We first characterized the reactions in the network to see if results of our stochastic model could reasonably be compared to results with standard ODE methods. A second order chemical reaction in the solvent phase consists of two steps: molecules encountering each other by diffusion followed by the molecules reacting with each other. If the encountering step takes a much longer time to occur than the reacting step, the reaction is diffusion-limited; otherwise, the reaction is activation-limited. Conventionally, an experimentally measured binding kinetic rate, *k*_*on*_, can be decomposed into an encounter rate, *k*_*enc*_, and an intrinsic activation rate, *k*_*a*_, using the following equation [41],

$$\frac{1}{k_{on}}=\frac{1}{k_{enc}}+\frac{1}{k_{a}}$$ (1)

For diffusion-limited reactions, *k*_*a*_ ≫ *k*_*enc*_ and *k*_*on*_ ≈ *k*_*enc*_; for activation-limited reactions, *k*_*on*_ ≈ *k*_*a*_ as diffusion is sufficiently fast. Given the diffusion coefficients for Ca^2+^ and CaM and the parameter values for Ca^2+^ and CaM reaction kinetics in S1 Table, we calculated the $\frac{k_{a}}{k_{on}}$ ratios and concluded that the reactions in the network belong to the activation-limited regime. This was also true if diffusion constants were reduced by half. Thus, this simple model resembles a well-mixed system and an ODE model will provide a good test standard.

We coded a deterministic ODE system in COPASI [42] and compared results with our stochastic model using the same kinetics. We found that the stochastic model and the ODE COPASI model exhibited similar time courses for all CaM binding state species. Slowing down Ca^2+^ diffusion did not significantly alter the time courses for the parameter values used (Fig 2C).

As another verification, we tested the steady state fraction of phosphorylation of CaMKII holoenzymes when they are modeled as multi-subunit complexes (Model 2, in S1 File). Michalski and Loew [22] derived an expression for the steady state fraction of phosphorylated subunits as a function of the number of subunits in a holoenzyme. This expression assumes a closed system where CaMKII holoenzymes are exposed to a saturating amount of fully loaded CaM molecules and subunit phosphorylation is allowed from a neighbor that has CaM bound but not from one that is also phosphorylated. With these conditions, the steady state fraction of phosphorylated subunits is $\frac{1}{2}$ for holoenzymes as dimers, $\frac{2}{3}$ for trimers, and approaches 1−*e*^−1^ for large subunit numbers. We built a model using a closed system with 1 μm × 1 μm × 1 μm geometry. The model contained 6000 CaM bound CaMKII subunits. CaMKII holoenzymes all had either 2, 3, 6 or 12 subunits. Consistent with the Michalski and Loew formula assumptions, an already phosphorylated subunit could not act as a kinase to phosphorylate its neighbor. As expected, we obtained steady state phosphorylation levels that depended on the number of the subunits in a holoenzyme as per the Michalski and Loew formula (S2 Fig).

Finally, using the same closed system, we tested the complete network and compared results with those obtained from a simulation implemented with a spatial Gillespie algorithm [20] (Model 3, S1 File). The two methods showed comparable results (S3 Fig). The results of all these tests suggest that our modifications of Smoldyn are working properly.

### Analysis of the Ca^2+^-CaM-CaMKII network with physiologically relevant Ca^2+^ influx

**Fig 3:**
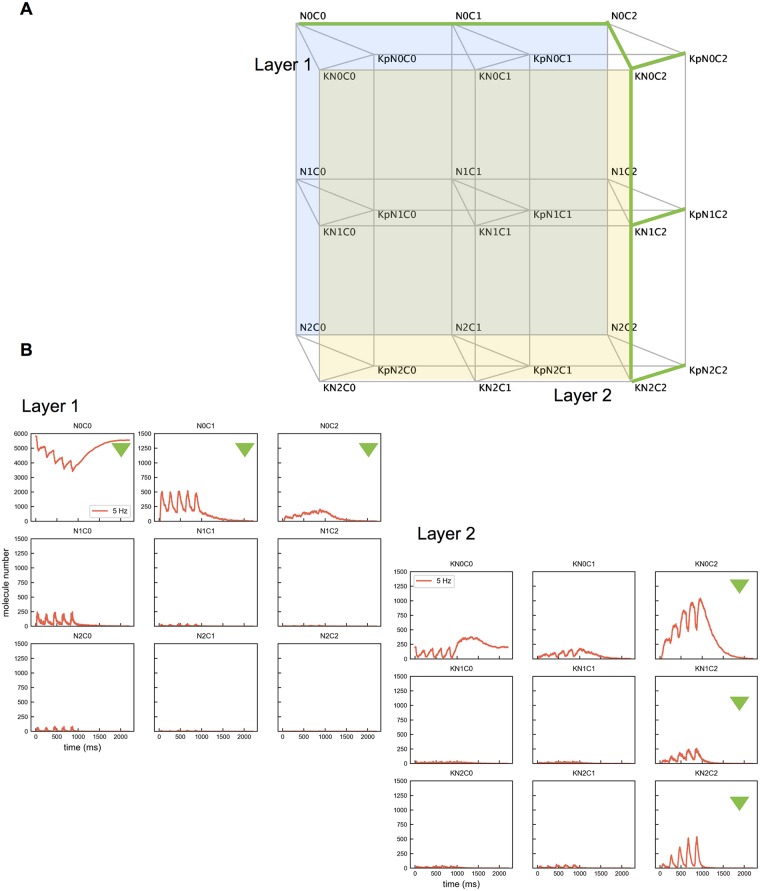
The complete Ca^2+^-CaM-CaMKII network. (A) Species are represented by vertices. Green edges mark the preferred pathway. The back layer (Layer 1, blue) represents interactions between Ca^2+^ and CaM. The front layer (Layer 2, yellow) represents interactions between Ca^2+^ and CaM attached to a CaMKII subunit. Note these interactions are bidirectional. (B) Molecule numbers of various CaM states with 5 Hz Ca^2+^ influx. Plots of Layer 1 and Layer 2 are displayed to provide a view of the preferred path. The species marked with green triangles constitute part of the preferred pathway. Note that only panel N0C0 in Layer 1 is on a scale from 0 to 6000. All the other panels have a scale from 0 to 1500.

To investigate how various CaM species contribute to the activation of CaMKII holoenzymes, we set up a prototype Ca^2+^-CaM-CaMKII network model. We were particularly interested in studying how network transitions involving partially loaded CaM species contribute to CaMKII activations, as this is not clearly understood. The prototype model comprises Ca^2+^-CaM-CaMKII interactions as shown in Fig 3A. In this network diagram, molecule species are represented as vertices and reactions are represented as edges.

CaM molecules and CaMKII holoenzymes with 6-subunit rings were initially uniformly distributed in a 1 μm × 1 μm × 2 μm box. As an initial condition, the box contained 6020 CaM molecules (5 μm) and 12036 CaMKII subunits (10 μm), within which 195 CaM molecules are bound to CaMKII. The initial state is at an equilibrium, which we carefully computed by running 5 simulation trials starting with the same numbers of CaM and CaMKII molecules. For simplicity, we did not consider resting intracellular Ca^2+^ as part of the steady state initial condition, as calculations showed its effect to be minor. Ca^2+^ influx was modeled as an entry from a single source at the top center of the box. A previously generated input file having a total of 5 Ca^2+^ bursts delivered at 5 Hz was used to provide Ca^2+^ influx during the simulation (see Methods).

We ran the simulation up to 2.2s, recorded the molecule numbers at every 1 ms (Fig 3B) and logged all reaction events. Using the events log, we counted the accumulated occurrences of each reaction type at every 10 ms, starting from the initial state until the Ca^2+^ bursts were finished. For a particular reaction, the number of occurrences during a time span is considered to be the net number of molecule state changes along the corresponding edge, i.e., the number of unbinding events is subtracted from the number of binding events. A negative number means that unbinding occurs more often than binding within the given time span.

**Fig 4:**
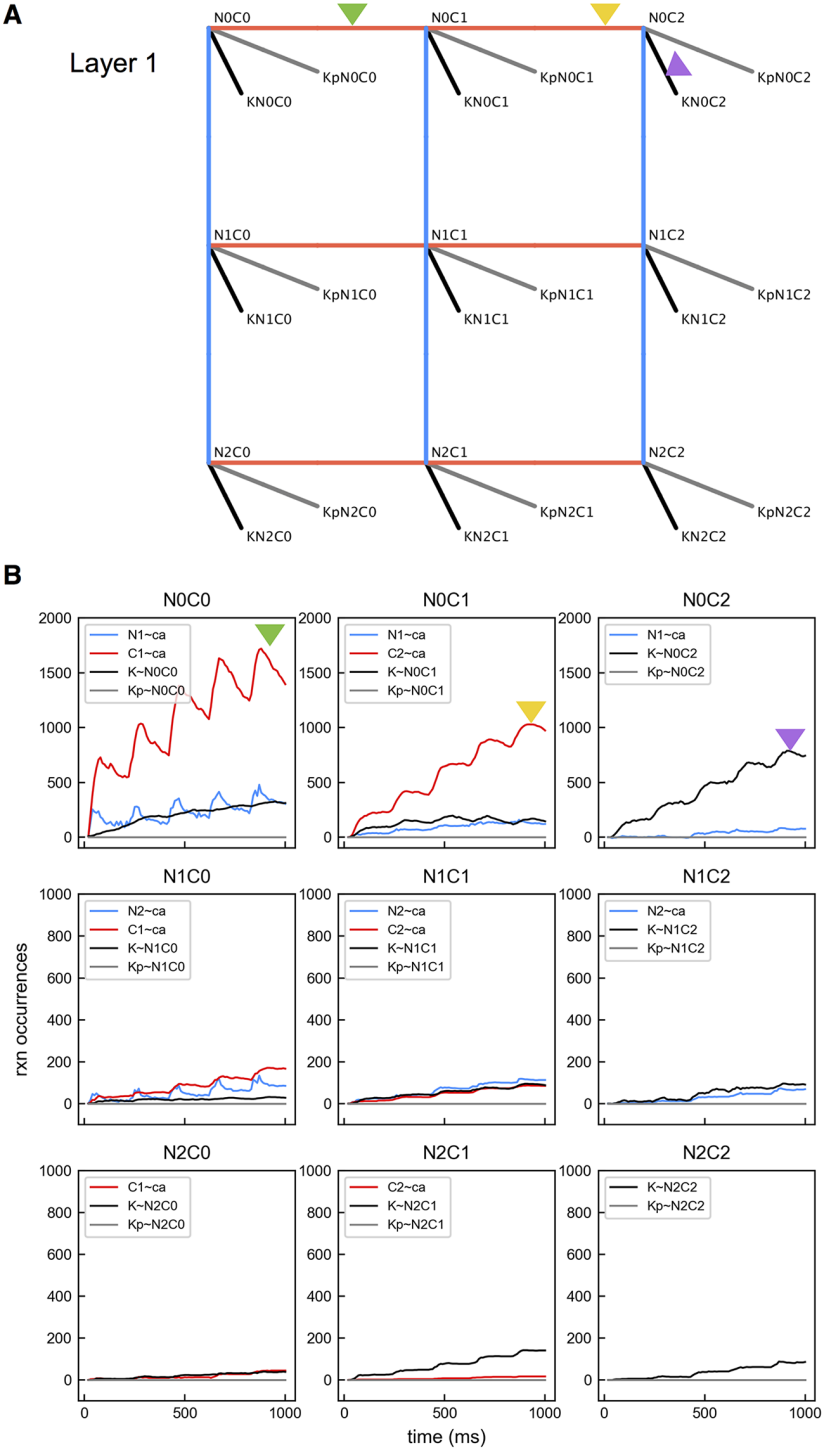
Layer 1 reactions. (A) Kinetic diagram illustrating possible reactions starting from Layer 1. For a CaM binding state, 4 types of bidirectional reactions are shown: interacting with a Ca^2+^ at an N site, with a Ca^2+^ at a C site, with an unphosphorylated CaMKII subunit and with a phosphorylated CaMKII subunit. Triangles indicate reactions involved in the preferred pathway starting from N0C0. (B) Accumulated reaction occurrences over time. Color code corresponds with the edge color in A. Colored triangles match plot lines with particular edges in A.

**Fig 5:**
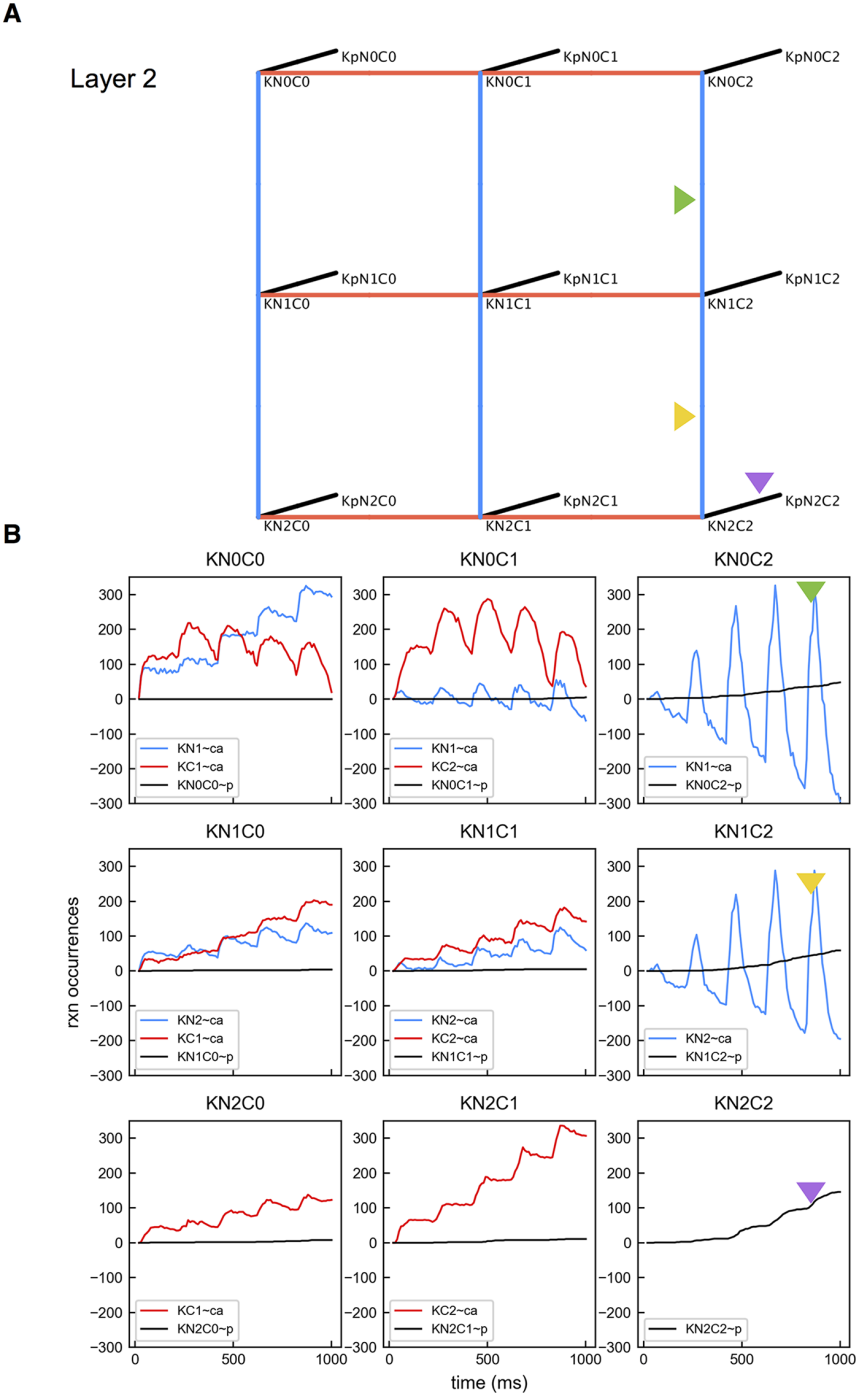
Layer 2 reactions. (A) Kinetic diagram illustrating possible reactions starting from Layer 2. For a CaM binding state, 3 types of reactions are shown: interacting with a Ca^2+^ at an N site, with a Ca^2+^ at a C site, and the bound CaMKII subunit becoming phosphorylated. Triangles indicate reactions involved in the preferred pathway. (B) Accumulated reaction occurrences over time. Color code corresponds with the edge color in A. Colored triangles match lines with particular edges in A.

We analyzed the edges of the Ca^2+^-CaM-CaMKII network (Fig 3A). We decomposed the network to 2 layers. The back layer (Layer 1) describes interactions between Ca^2+^ and the 9 states of CaM (NxCy, x,y = 0,1,2). The front layer (Layer 2) is for reactions of Ca^2+^ with CaM attached to unphosphorylated CaMKII (KNxCy). Starting in Layer 1, there are up to 4 possible binding reactions for each CaM species to change state (Fig 4A): binding a Ca^2+^ at a C site (denoted as Cx∼ca), binding Ca^2+^ at an N site (denoted as Nx∼ca), binding to an unphosphorylated CaMKII subunit (denoted as K∼NxCy) or binding to a phosphorylated CaMKII subunit (denoted as Kp∼NxCy). Likewise, in Layer 2, there are up to 3 possible binding reactions for each CaMKII bound CaM species to change state (Fig 5A): binding a Ca^2+^ at a C site (denoted as KCx∼ca), binding a Ca^2+^ at an N site (denoted as KNx∼ca), and the CaMKII bound with the CaM becoming phosphorylated (denoted as KNxCy∼p).

For each CaM state, we counted the accumulated occurrences of the possible reaction types. Results are shown in Fig 4B. The plot reveals a preferred pathway for CaM and CaMKII state transitions as labeled in Fig 3A. Starting as apoCaM, a CaM molecule tends to bind a Ca^2+^ ion on the C1 site and then on the C2 site, entering the N0C2 state. CaM in the N0C2 state has a strong preference to enter Layer 2 by binding to a CaMKII subunit (Fig 4B, the black line in panel N0C2), likely followed by the phosphorylation of the bound CaMKII subunit (Fig 5B, the black line in panel KN0C2). Once bound to a CaMKII subunit, additional Ca^2+^ ions bind to CaM more quickly. In this scenario, it is rare for CaM to become fully bound with Ca^2+^ ions before binding with a CaMKII subunit. Nevertheless, phosphorylation of CaMKII subunits occurs most often when subunits are bound with fully loaded CaM, but still often occurs with CaM having C sites loaded, KN0C2 and KN1C2, as shown in Fig 5B. We note that the way the preferred pathway is chosen at each vertex is a consequence of reaction affinities as given in S1 Table and these were chosen to be consistent with experimental studies and values in the previous modeling work [19–21]. For example, even though N sites bind Ca^2+^ faster, the C sites have higher affinity making Ca^2+^ binding to C sites preferred.

**Fig 6:**
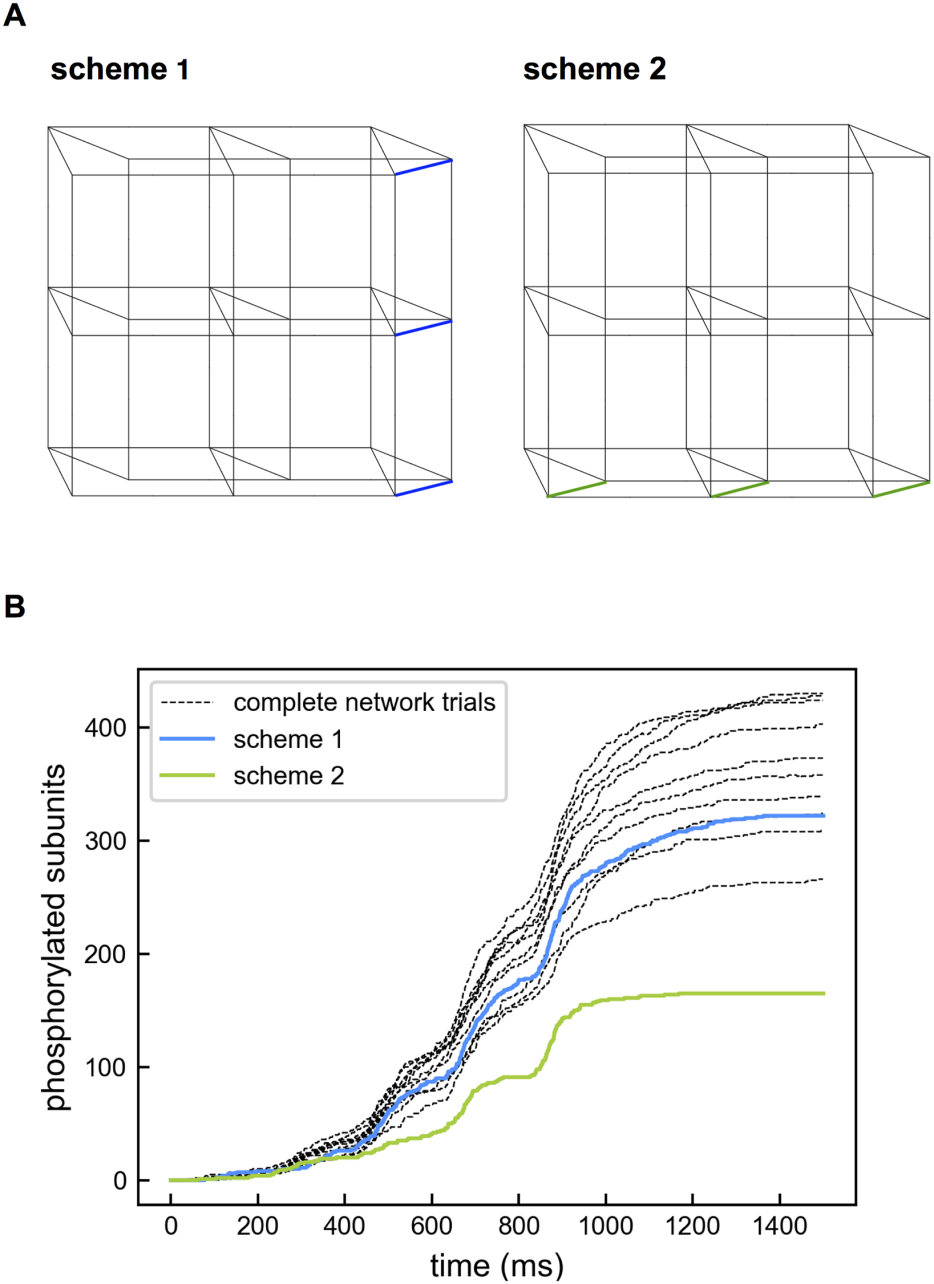
Reaction scheme 1 and 2 adapted from the complete network. (A) In reaction scheme 1, phosphorylation was allowed only from CaMKII bound with NxC2 (blue edges). In reaction scheme 2, phosphorylation was allowed only from CaMKII bound with N2Cx (green edges). (B) Comparison of phosphorylation levels in the complete network (10 individual trials), scheme 1 and scheme 2.

To confirm the critical role of NxC2 (x = 0,1,2) CaM in activation and phosphorylation of CaMKII, we set up two modified reaction schemes (Fig 6A). In Scheme 1, only CaMKII subunits bound with NxC2 are allowed to become phosphorylated. In Scheme 2, phosphorylation is allowed only for subunits bound with N2Cx. Note that the phosphorylation rates in the two schemes are equivalent, i.e., *k*_*on*_ of reaction KN2C0∼p is the same as that of KN0C2∼p, and the *k*_*on*_ of KN2C1∼p equals that of KN1C2∼p. Not surprisingly, the two schemes give rise to different phosphorylation levels as shown in Fig 6B. Scheme 1 performs slightly worse than the whole network, whereas Scheme 2 produces a much lower phosphorylation level. Therefore, edges of the network are not equally involved in CaMKII phosphorylation.

### Changes in CaMKII activation frequency dependence and physiologically relevant factors

A classic experiment by DeKoninck and Schulman [43] showed that *in vitro*, CaMKII holoenzyme activation is sensitive to the frequency of Ca^2+^-CaM pulses. A recent study [44] also showed that *in vivo* glutamate uncaging frequency affects CaMKII activation in dendritic spines. Numerous modeling studies have also reported a dependence of CaMKII activation on the frequency of Ca^2+^ signals [16, 17, 21, 22]. To confirm this CaMKII activation pattern, we tested our 6-subunit CaMKII model using previously generated 10 Hz and 5 Hz Ca^2+^ influx files. The total number of Ca^2+^ ions entering was comparable in the two cases (40433 ions with 5 Hz vs. 40435 ions with 10 Hz).

**Fig 7:**
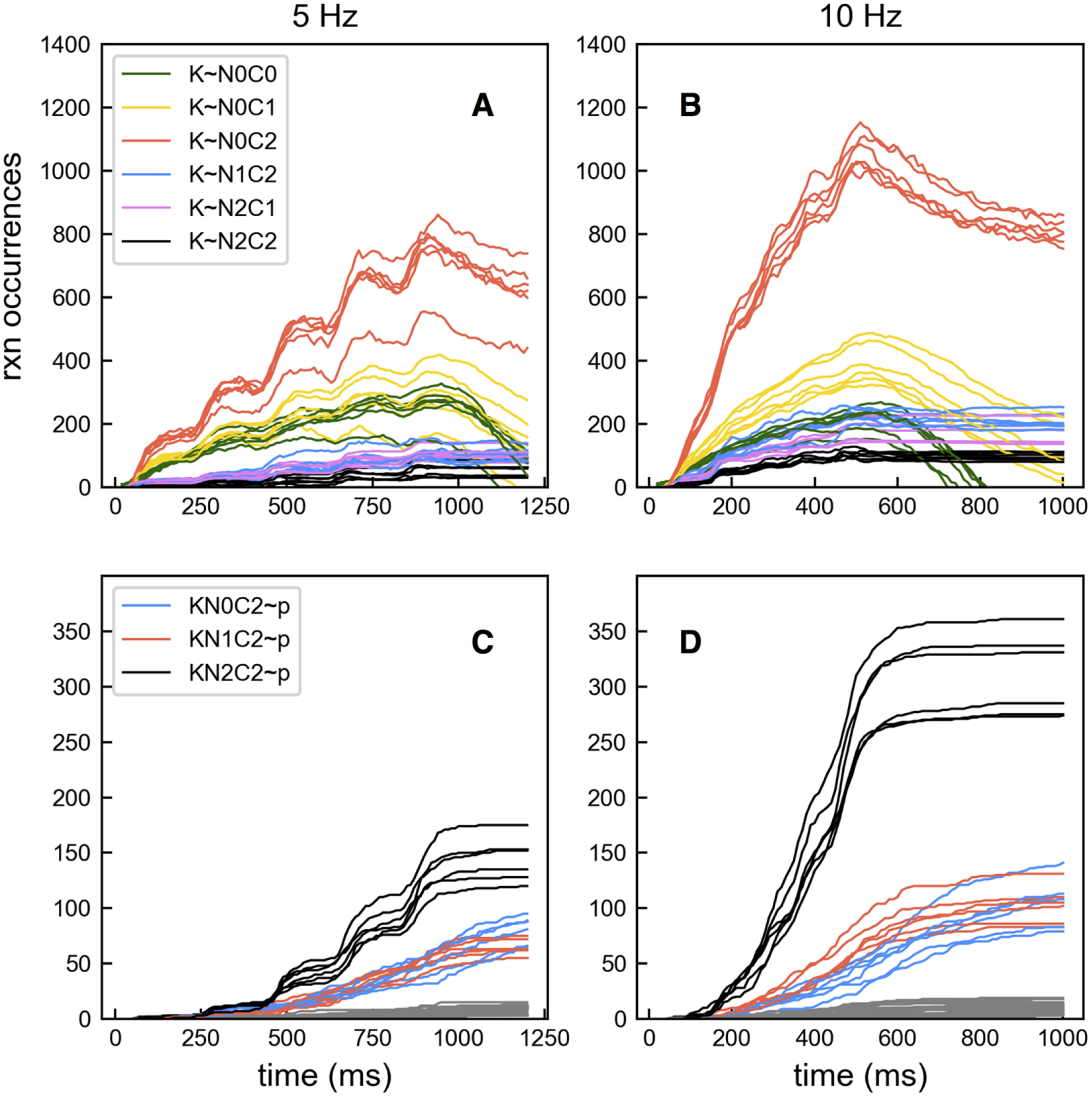
Effects of 10 Hz and 5 Hz Ca^2+^ input on reaction occurrences. (A) Accumulated reaction occurrences generated from 6 trials for CaM CaMKII binding reactions in the presence of 5 Hz Ca^2+^ input. (B) The same as in A but for 10 Hz Ca^2+^ input. (C) Accumulated reaction occurrences for CaMKII phosphorylation reactions in the presence of 5 Hz Ca^2+^ input. Phosphorylation reactions other than KNxC2∼p are shown in grey and are not individually labeled. (D) The same as in C but for 10 Hz Ca^2+^ input. For a different view of these data, see S6 Fig.

We examined the reaction occurrences over time and noticed that, compared to 5 Hz input, the 10 Hz input results in more rapid and more overall bindings between N0C2 CaM and CaMKII (Fig 7A and 7B, also see S6 Fig), and consequently, more autophosphorylation. More bindings between N0C2 CaM and CaMKII is to be expected because higher frequency means more intensive Ca^2+^ input which drives CaM to travel through the preferred pathway (Fig 3A). This leads to more N0C2 available to bind to CaMKII. More autophosphorylation is primarily due to increasing occurrences of KN2C2∼p reactions (Fig 7C and 7D) because the additional KN0C2 binds Ca^2+^ at higher affinity leading to state KN2C2 that has the highest rate of autophosphorylation. Therefore, the frequency effect is inherent in the binding properties among Ca^2+^, CaM and CaMKII.

Interestingly, the effect of frequency is not the same for all CaMKII CaM binding interactions. For example, the net bindings between N0C1 CaM and CaMKII increased with 10 Hz input while bindings between apoCaM and CaMKII decreased. To understand this note that with high frequency Ca^2+^ input, [Ca^2+^] is higher and more N0C1 forms. With more N0C1 available, there will be more binding of N0C1 with CaMKII. Hence the yellow line (K∼N0C1) is higher in Fig 7B (10Hz) than in Fig 7A (5Hz). In addition, there will be more Ca^2+^ binding to KN0C0, forming KN0C1, and this means that mass action kinetics will drive N0C0 to bind with CaMKII to replace the KN0C0 that transitioned to KN0C1. However, more N0C1 formed from N0C0 with high frequency input means that there is less N0C0 available to drive the K∼N0C0 reaction. As a result, the green line (K∼N0C0) is lower in Fig 7B (10Hz) than in Fig 7A (5 Hz).

**Fig 8:**
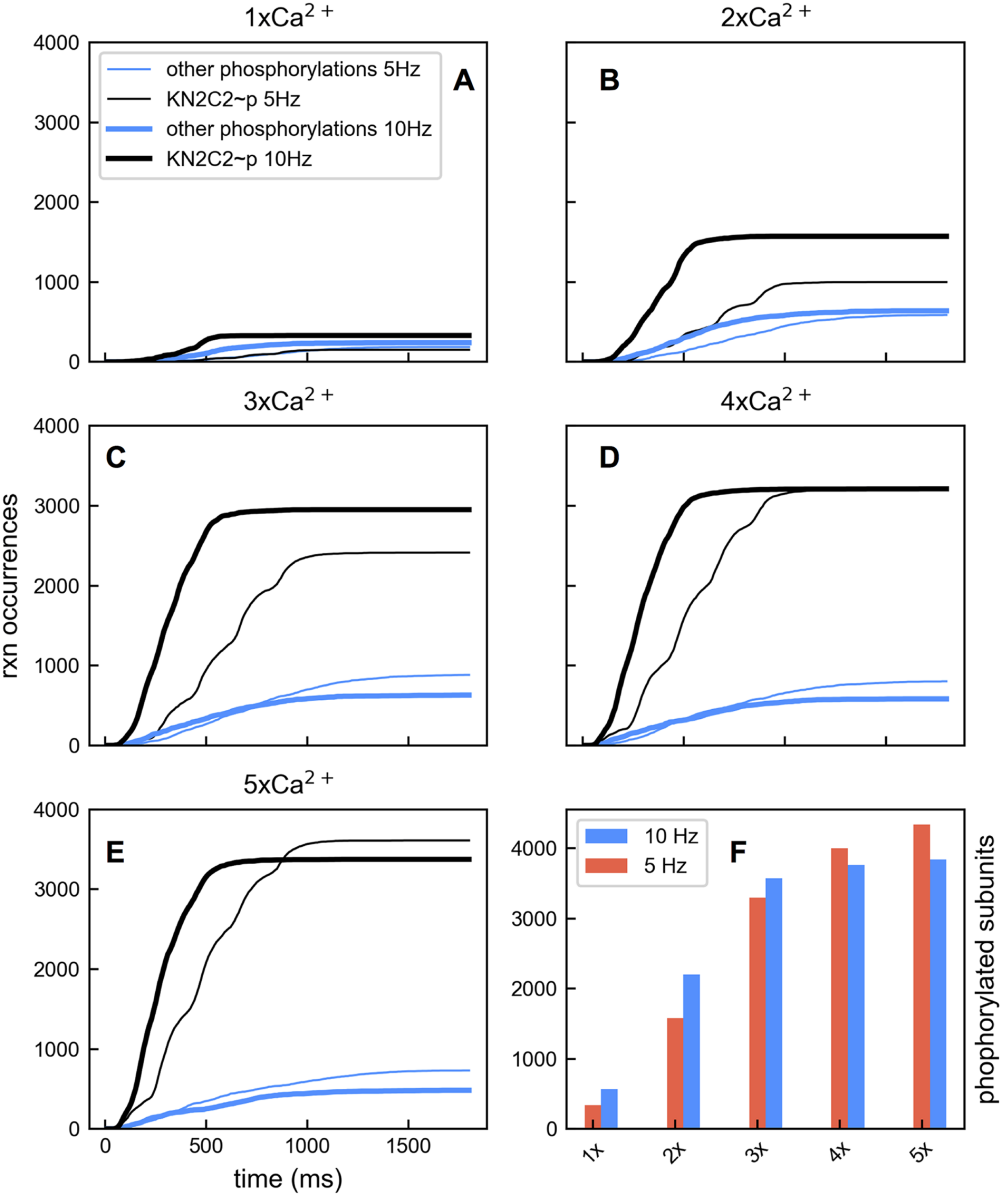
Effects of increased Ca^2+^ influx on frequency dependence. (A-E) Accumulated reaction occurrences for phosphorylation of CaMKII subunits bound with N2C2 CaM (black lines) and all other NxCy CaM summed together (blue lines) are shown for 5 Hz (thin lines) and 10 Hz (thick lines) input with different levels of Ca^2+^ influx. (F) Summary of phosphorylation levels in the presence of increasing amounts of Ca^2+^ delivered at 5 Hz and 10 Hz. The advantage of 10 Hz input for phosphorylation is reversed as Ca^2+^ influx increases.

However, the frequency effect can be reversed by providing a saturating amount of Ca^2+^. In Fig 8, we increased the Ca^2+^ channel number to allow more Ca^2+^ influx per action potential pulse. The total Ca^2+^ influx was again comparable for 5 Hz and 10 Hz input. As Ca^2+^ influx was increased, the network produced more phosphorylated CaMKII subunits for both input frequencies, but the difference in phosphorylation level between 5 Hz and 10 Hz input diminished. Eventually, the 10 Hz input became saturating and the network generated less phosphorylation with 10 Hz than with 5 Hz input (Fig 8F).

**Fig 9:**
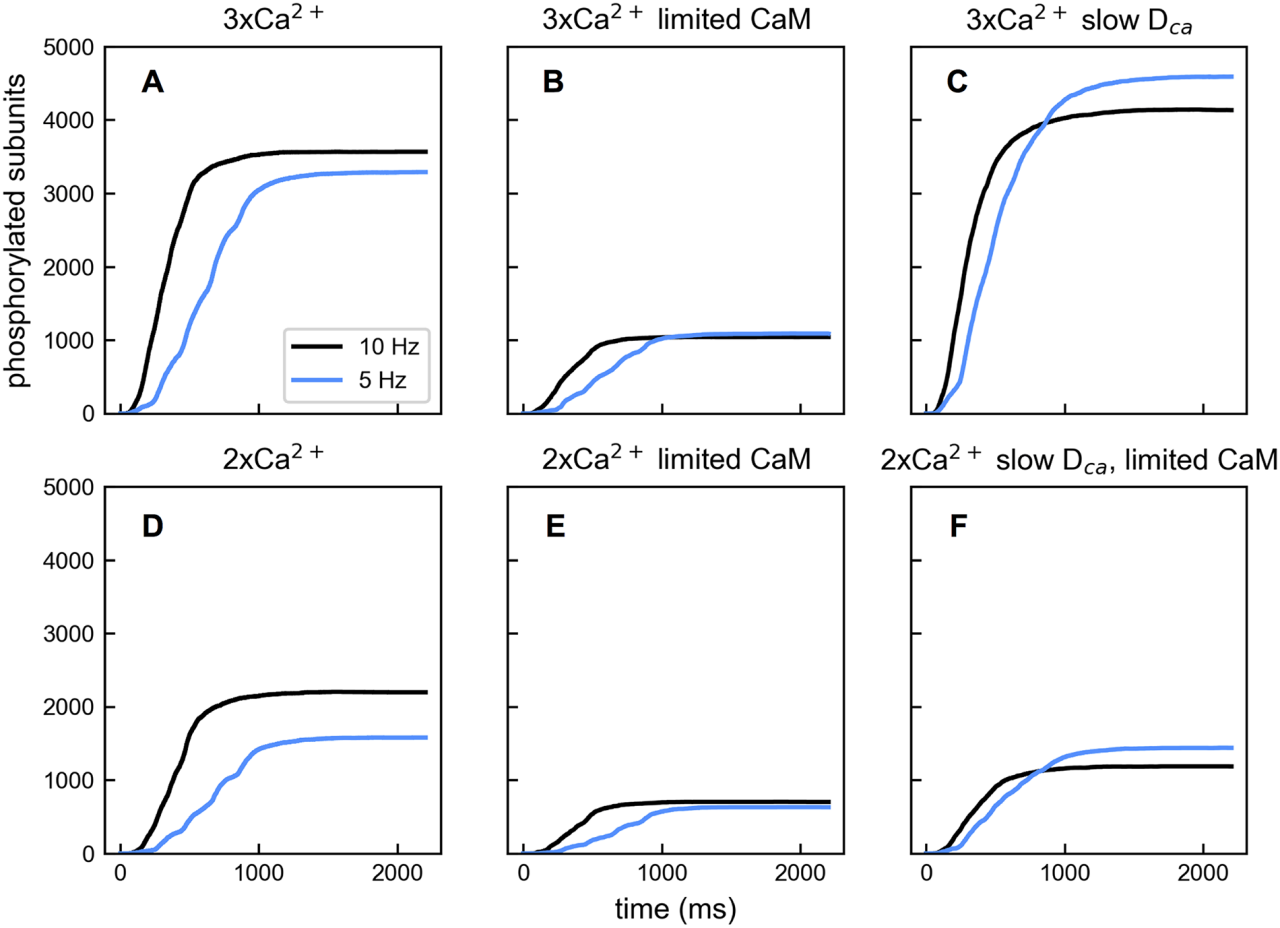
Effects of CaM availability and Ca^2+^ diffusion on frequency dependence. (A) With 3× Ca^2+^ influx, phosphorylated CaMKII subunits in the presence of 5 Hz (blue) and 10 Hz (black) Ca^2+^ input. (B) The same as in A except that the total CaM available in the system is reduced from 5 μm to 2.5 μm. (C) The same as in A except that Ca^2+^ diffusion is slowed to 1.1 × 10^−6^ cm^2^ s^−1^. (D) The same as in A except that 2× Ca^2+^ influx is provided. (E) The same as in D except that the total CaM available in the system is reduced from 5 μm to 2.5 μm. (F) The same as in D except with both slowed Ca^2+^ diffusion and reduced the amount of CaM.

This reversal in frequency preference occurs at lower levels of Ca^2+^ input when available CaM is limited or Ca^2+^ diffusion is slowed (Fig 9). Notice that in the model Ca^2+^ can diffuse out of the reaction volume. As a result, a lower diffusion constant effectively means a higher level of Ca^2+^. To demonstrate, we first compared simulations where the amount of CaM in the system was reduced from the default concentration of 5 μm to 2.5 μm. Limiting CaM reduces phosphorylation for a given Ca^2+^ input at both frequencies (compare Fig 9A and 9B), but the difference in phosphorylation levels between the two frequency conditions is smaller. We see slightly more phosphorylation with 5 Hz input than 10 Hz input with the 3× Ca^2+^ input condition. With less CaM present, the Ca^2+^ influx has a better chance to saturate the available CaM allowing the frequency dependence to reverse at a lower influx level. We then compared simulations with default (2.2 × 10^−6^ cm^2^ s^−1^) and slowed (1.1 × 10^−6^ cm^2^ s^−1^) Ca^2+^ diffusion coefficients, noting that the slowed diffusion condition does not change the activation-limited regime of the network. Slowed diffusion results in a dramatic increase of CaMKII phosphorylation regardless of Ca^2+^ influx level and input frequency (compare Fig 9A and 9C). Here the frequency preference for 10 Hz input also reverses at the 3× Ca^2+^ input condition. This occurs because slow Ca^2+^ diffusion allows the input to become sufficiently saturating at a lower level of Ca^2+^ influx and this leads to the frequency preference change. Finally, frequency dependence reverses dramatically with merely twice the baseline amount of Ca^2+^ influx when slow diffusion and limited CaM are combined (Fig 9D–9F).

**Fig 10:**
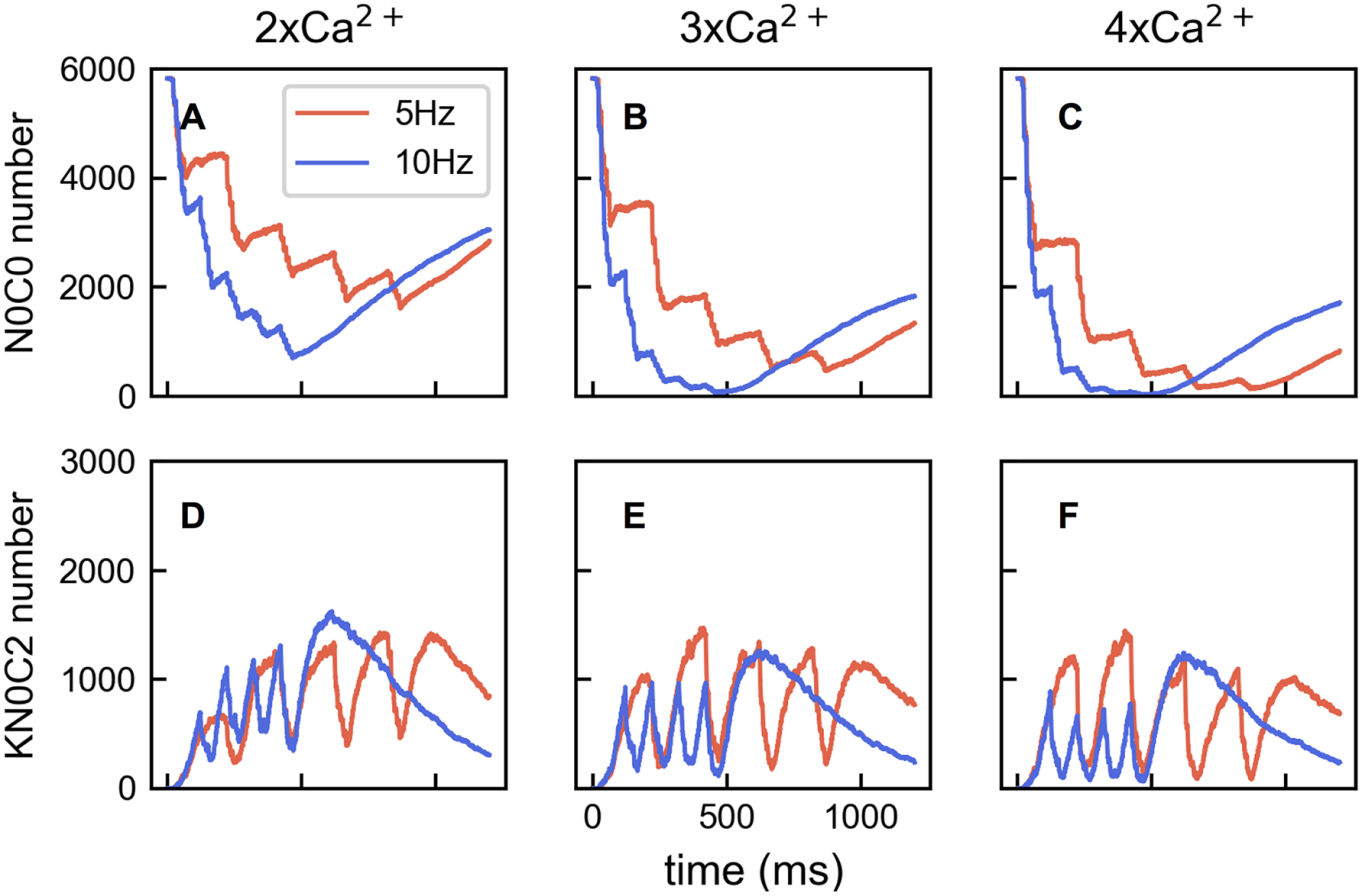
ApoCaM depletion and KN0C2 number as Ca^2+^ influx increases. (A) With 2× Ca^2+^ input, time courses of the amount of apoCaM (N0C0). Red lines are for 5 Hz input, and blue lines are for 10 Hz input. (B) The same as in A except that 3× Ca^2+^ influx is given. (C) The same as in A except that 4× Ca^2+^ influx is given. (D-F) The same as in A-C except that KN0C2 molecule numbers are plotted.

To understand why frequency dependence changes, we examined what was happening in the full Ca^2+^-CaM-CaMKII network starting with Ca^2+^ binding to apoCaM and apoCaM availability. It is important to note that simulations with 10 Hz input and 5 Hz input had virtually the same total Ca^2+^ entering the volume. With 3× Ca^2+^ influx, 10 Hz bursts deplete apoCaM, but 5 Hz bursts do not (Fig 10B). With 4× Ca^2+^ influx, it takes 4 bursts of influx to deplete apoCaM in the 5 Hz condition but only 3 with 10 Hz (Fig 10C). When apoCaM molecules are depleted, Ca^2+^ must bind elsewhere in the network and this causes deviations from the preferred path as described below. One consequence is that the number of KN0C2 molecules formed is higher with 5 Hz input (Fig 10E and 10F, red lines) than with 10 Hz input (Fig 10E and 10F, blue lines). This leads to more phosphorylation with 5 Hz than 10 Hz input for the 4× Ca^2+^ influx case because of the longer inter-burst interval, which allows more relief from apoCaM depletion. Thus Ca^2+^ can bind to apoCaM and subsequent reactions along the preferred path can occur. The frequency dependence reversal for reduced CaM availability and slowed Ca^2+^ diffusion can also be explained in terms of CaM saturation. For a given Ca^2+^ input apoCaM depletion will occur faster if there is less to begin with and also if Ca^2+^ diffusion is slowed, which will allow more chances for Ca^2+^ and CaM to interact before Ca^2+^ leaves the system.

**Fig 11:**
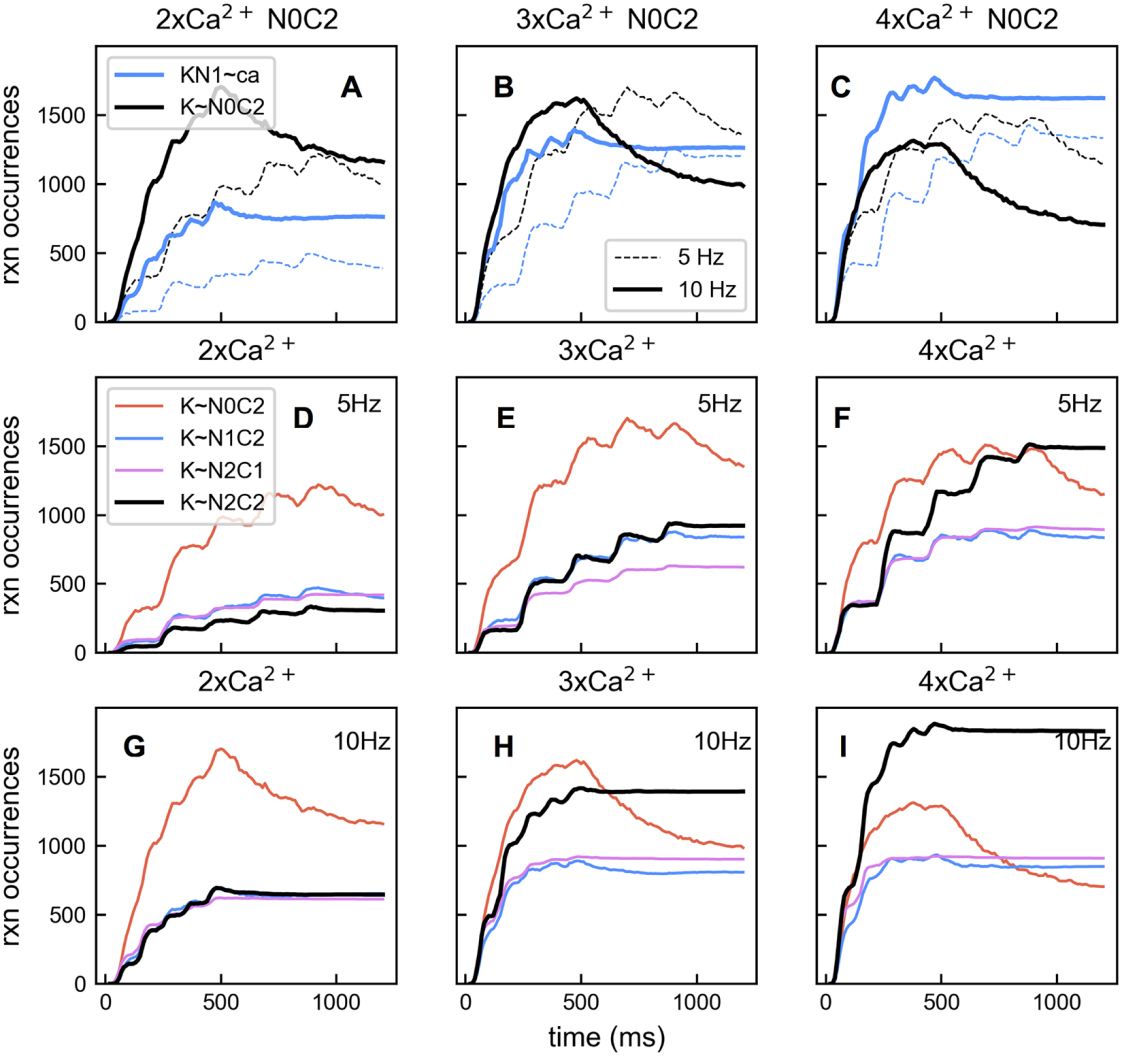
Change of pathway choice as Ca^2+^ influx increases. (A) With 2× Ca^2+^ input, reaction occurrences of CaM N0C2 binding Ca^2+^ at the N1 site (blue) and directly binding with CaMKII (black). Thick lines are for 10 Hz input, and thin lines are for 5 Hz input. (B) The same as in A except that 3× Ca^2+^ influx is given. (C) The same as in A except that 4× Ca^2+^ influx is given. (D-F) Accumulated reaction occurrences for major CaM CaMKII binding reactions with varying amount of total Ca^2+^ influx from 2×, 3× to 4×, with Ca^2+^ input delivered at 5 Hz. (G-I) The same as in D-F except that Ca^2+^ input is delivered at 10 Hz.

**Fig 12:**
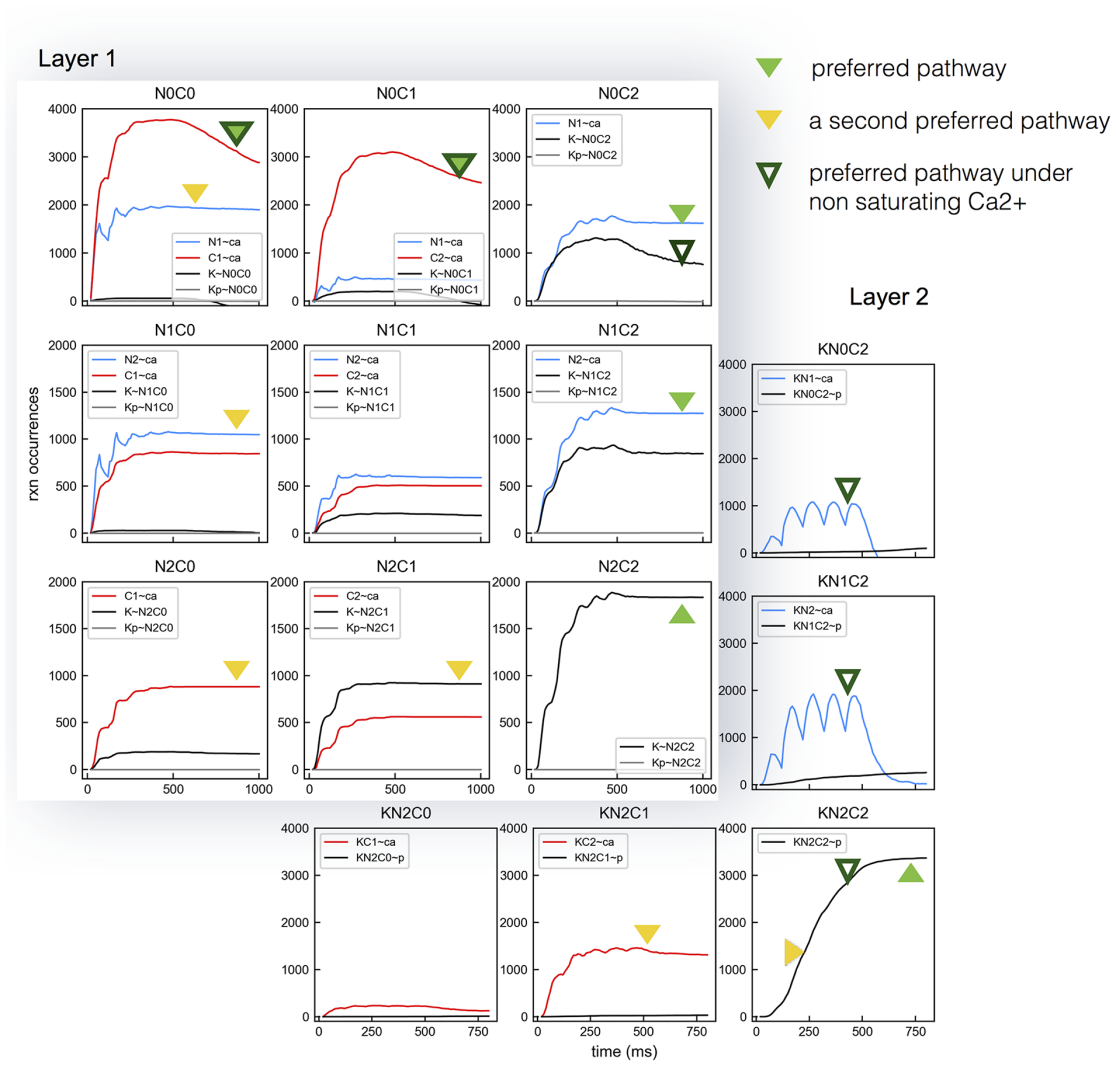
Deviations in the preferred pathway with 5× Ca^2+^ influx at 10 Hz. Accumulated reaction occurrences for CaM species in Layer1 and Layer 2 are shown. The new preferred pathway (marked by solid green triangles) is different from the one previously observed with a non-saturating Ca^2+^ influx in Figs 4 and 5 (marked by hollow green triangles). Also note a second preferred pathway that travels through N2C1 (marked by yellow triangles).

We found that there were deviations from the previously observed preferred pathway when saturating Ca^2+^ input is provided, particularly when CaM molecules are in the N0C2 state. In the presence of moderate Ca^2+^ influx (Fig 11A and 11B) CaM molecules follow the preferred pathway: the net bindings between N0C2 and CaMKII (K∼N0C2) occur more often than the bindings of Ca^2+^ ions to the N1 site of N0C2 molecules (N1∼ca). CaMKII binding with N0C2 dominates all types of CaMKII CaM interactions (Fig 11D, 11E and 11G). However, when Ca^2+^ influx becomes saturating, bindings of Ca^2+^ ions on the N1 site of N0C2 molecules start to dominate over the bindings between N0C2 and CaMKII (Fig 11C). CaM molecules tend to stay in Layer 1 until they get fully loaded with Ca^2+^. As a result, CaMKII binding with N2C2 increases and becomes dominant over all other CaMKII CaM interactions (Fig 11F, 11H and 11I). This gives rise to an altered preferred pathway (Fig 12 solid green triangles). It is worth noting that the net bindings between CaMKII and N1C2 and N2C1 also rise as Ca^2+^ input becomes saturating (Fig 11D–11I, blue lines and pink lines). Interestingly, when we examined the accumulated reaction occurrences with 5× Ca^2+^ influx at 10 Hz, we noticed that K∼N2C1 constitutes a second preferred pathway (Fig 12 yellow triangles), which starts with N1∼ca reactions from apoCaM followed by N2∼ca, C1∼ca, and K∼N2C1 in Layer 1, and KC2∼ca and KN2C2∼p in Layer 2. In short, a saturating amount of Ca^2+^ forces CaM species to traverse states in a way that deviates from the preferred pathway observed with a moderate amount of Ca^2+^. These deviations are driven by different amounts of reactants.

**Fig 13:**
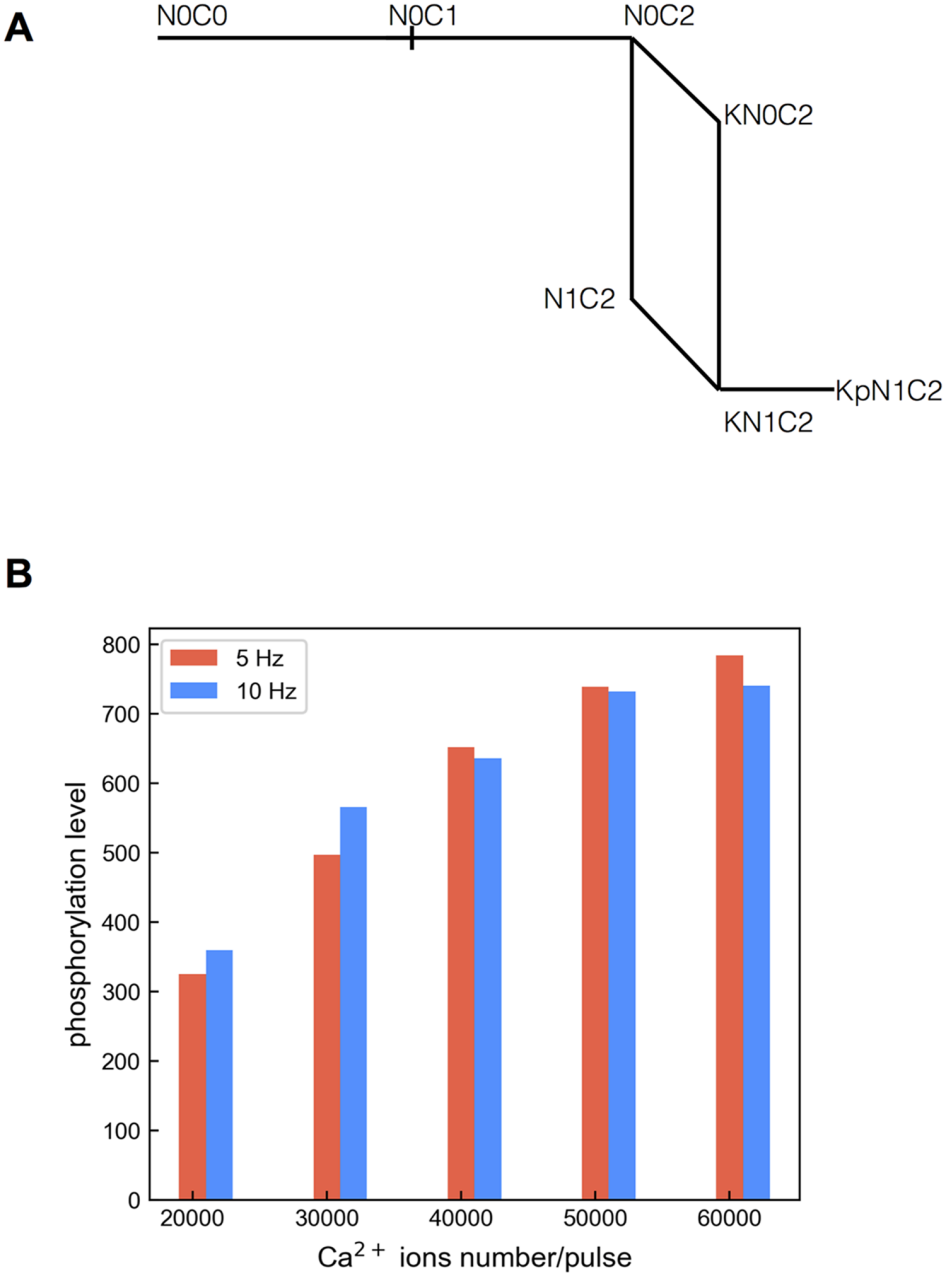
The effect of saturating Ca^2+^ on reversing frequency dependence demonstrated using a reduced network. (A) The reduced reaction network derived from the complete network. CaMKII is modeled as a monomer and undergoes phosphorylation at an arbitrary rate 1 s^−1^ as long as it is bound with a CaM N1C2. (B) Summary of phosphorylation levels in the presence of varying Ca^2+^ ions per pulse from 20,000 ions/pulse to 50,000 ions/pulse delivered at 5 Hz and 10 Hz.

To validate our conclusion about the pathway decision change, we used a reduced reaction network to capture the observed frequency preference reversal (Fig 13). The reduced network is derived from the initial steps in the whole Ca^2+^-CaM-CaMKII network. We used the original Smoldyn to simulate the simple network, to demonstrate that the reversal of frequency preference is not an artifact of our modified simulator but is inherent to the network itself. We used 5 pulses of instantaneous Ca^2+^ release as the input and varied the amount of Ca^2+^ influx per pulse. The CaMKII molecules are modeled as monomers with an arbitrary phosphorylation rate of 1 s^−1^. As expected, the reversal of frequency preference can be qualitatively captured by the simple reaction scheme (Fig 13A). The 10 Hz stimulus becomes saturating and fails to generate more phosphorylation than 5 Hz when Ca^2+^ influx reaches 50,000 ions per pulse (Fig 13B).

### The structural organization of CaMKII subunits may affect the activation level

Recent work on CaMKII holoenzyme structure suggests that how subunits are organized can affect the activation of the holoenzyme. In particular, whether subunits are arranged in a compact or an extended way can change the accessibility of CaM to CaMKII. It is known that the structural arrangement is related to a linker region between a subunit’s kinase domain and the holoenzyme central hub. Bayer et al. [45] examined splice variants of *β*-CaMKII, which have identical kinase domains yet different linker lengths. These variants responded to Ca^2+^ oscillations differently, even though they showed no response difference to prolonged Ca^2+^-CaM input. In particular, for Ca^2+^ pulse input, the variants with longer linker length exhibited a higher autophosphorylation rate. Another study by Chao et al. [46] also indicated that the linker length affects the capability of a subunit’s kinase domain to undock from the central hub. Undocking helps the subunit to be released from an autoinhibited state. Thus a longer linker is expected to keep the kinase domain further away from the central hub, allowing a better chance for Ca^2+^-CaM to access the binding site.

**Fig 14:**
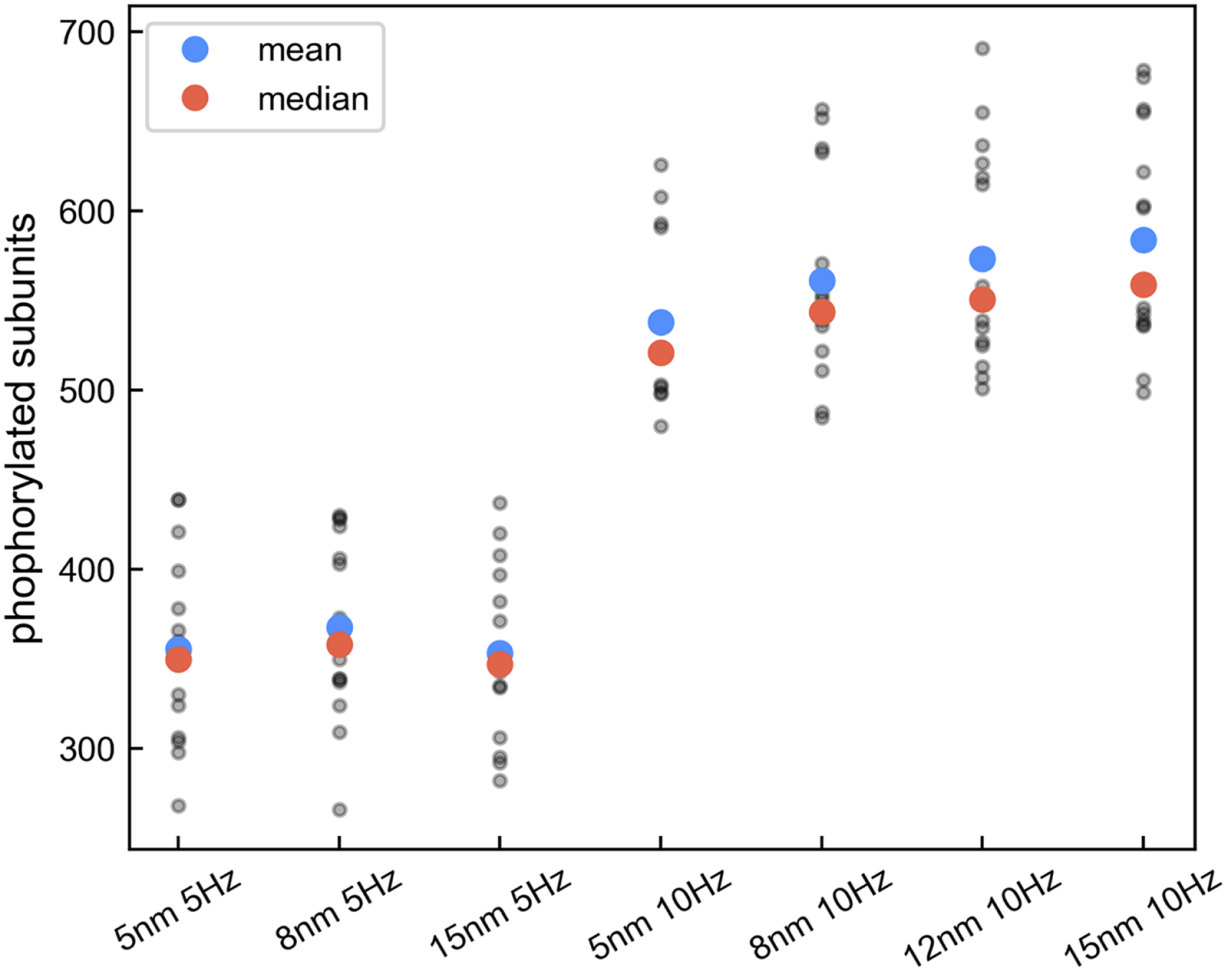
Effects of holoenzyme size on CaMKII phosphorylation. Phosphorylation levels from individual trials as a function of the holoenzyme radius and Ca^2+^ input frequency. Each radius condition contains 15 trials. For the trials with 10 Hz input, a one-way ANOVA test (F = 1.806, p = 0.157) was performed. Despite no statistically significant difference among radius conditions, a regression analysis shows a steady upward trend (slope = 4.359, p = 0.025) with statistical significance in phosphorylation level as the holoenzyme radius increases.

In our prototype model, each holoenzyme subunit has a distinct physical location and is separated by 8 nm from its neighbors. Studies suggest that a typical one-ring holoenzyme radius is 5 nm to 8 nm [46, 47]. To examine the effect of a longer linker length on CaMKII activation, we varied the radius of a one-ring 6-subunit holoenzyme from 5 nm to 15 nm. Neighbor subunits are equally spaced at the same distance as the holoenzyme radius. For each radius condition, we ran 15 simulation trials using the default Ca^2+^ influx delivered at 5 Hz and 10 Hz respectively. As shown in Fig 14, the effects of holoenzyme radius on phosphorylation level are related to the Ca^2+^ input frequencies. With 10 Hz pulses, phosphorylation increases steadily as the holoenzyme radius grows from 5 nm to 15 nm. This result suggests that linker length may affect Ca^2+^-CaM binding to CaMKII and subsequently affect phosphorylation levels, depending on the Ca^2+^ input conditions.

## Discussion

In this study, we present an efficient approach to simulate multi-subunit molecules with detailed kinetics in reaction-diffusion networks. Our approach is an adaptation of Smoldyn [37, 38], a particle-based stochastic simulator that allows reaction and diffusion to be simulated in spatially heterogeneous environments. Our adaptation adds new data structures to Smoldyn to describe reactions at the binding site level, leading to an efficient solution of the problem of combinatorial explosion inherent in models with multi-subunit molecules [24]. We also introduced an intuitive approach to analyze the pathway choices of a network based on the reaction history of a simulation, allowing us to grasp insights quickly about how reactions proceed in a large network. In traditional analyses, the numbers of each molecular species are examined to resolve the dynamics of the network. However, this procedure is indirect at best when it comes to drawing insights about network behavior. Furthermore, being able to track reaction history of a system is not possible in deterministic simulations. Here we were able to count the number of times a reaction occurs easily and we demonstrated that this reaction history can be highly informative and can allow us to see clearly how the favored network pathway may change with different input conditions.

### Insights from the detailed Ca^2+^-CaM-CaMKII network model

We used the modified Smoldyn simulator and reaction history information to obtain several insights into the Ca^2+^-CaM-CaMKII reaction network. First, under physiological conditions when Ca^2+^ influx is low to moderate, CaM molecules partially loaded with Ca^2+^ are important for CaMKII activation. In particular, reaction history shows that CaM molecules that have 2 Ca^2+^ ions attached on the C lobe (in particular species N0C2, but also N1C2 and N2C2) preferentially bind to CaMKII subunits before adding additional Ca^2+^ ions to the N lobe. This is consistent with the predominant pathway hypothesis suggested by Pepke et al. [21] as well as with experimental work by Shifman et al. [29]. Nevertheless, phosphorylation was found to occur primarily from CaMKII bound with CaM fully loaded with Ca^2+^(KN2C2) and higher frequencies of low to moderate Ca^2+^ input resulted in more CaMKII phosphorylation.

Second, while CaMKII activation is known to be sensitive to the frequency of Ca^2+^ signals [43], we found that the frequency dependence is reversed with strong Ca^2+^ signals, with more CaMKII subunit phosphorylation seen at 5 Hz input than at 10 Hz. Reaction history shows that this occurs because of a depletion of apoCaM, resulting in a change in the preferred pathway for CaMKII activation. Specifically, CaM with 2 Ca^2+^ ions on the C lobe now becomes more likely to bind Ca^2+^ on the N lobe than to bind with CaMKII. The change of pathway choice affects how the network is tuned to a particular Ca^2+^ input frequency. The fact that the strength of the Ca^2+^ signal matters is important because many experiments that study frequency dependence are done in conditions where CaM is saturated with Ca^2+^ and this is not the typical situation encountered by the cell.

Third, we found that factors such as CaM availability and Ca^2+^ diffusion can also affect the frequency dependence of CaMKII activation by Ca^2+^ signals, also by changing the preferred pathway for CaMKII activation. A limited amount of CaM makes the given Ca^2+^ input more likely to saturate available CaM on both lobes before binding to CaMKII. Similarly, slow Ca^2+^ diffusion allows more extensive interactions between Ca^2+^ and CaM thus making it more likely for a given amount of Ca^2+^ input to become saturating. Experimental studies suggest that the number of freely diffusible CaM molecules is highly limited *in vivo* [48–50] and limited CaM further implies a regulatory role for the many endogenous CaM-binding proteins [51]. A limited amount of CaM or a slowed Ca^2+^ diffusion may permit enough Ca^2+^ to bind to available CaM and allow CaMKII activation to occur at a lower frequency or a lower strength Ca^2+^ signal.

Fourth, it is known that intracellular crowding and spatial homogeneity can slow down molecule diffusion. For example, a recent biophysical study [52] suggests that the diffusion of Ca^2+^ ions can be reduced by ten times in a nanodomain around the Ca^2+^ channel mouth. We believe that such a restriction in Ca^2+^ diffusion may have substantial effects on CaMKII phosphorylation and the frequency dependence. For example, depending on the size of the nanodomain, slow Ca^2+^ diffusion in a nanodomain can potentially result in a localized Ca^2+^ signal with sufficient strength to activate CaMKII and downstream cascading proteins.

Finally, we demonstrated that holoenzyme size is a possible means to affect the level of phosphorylation, depending on the Ca^2+^ influx. We increased the size of the holoenzyme by changing the distance between neighboring subunits and found that with 10 Hz pulses the corresponding phosphorylation levels of the network increased. This is consistent with the idea that the configuration of a holoenzyme, whether compact or extended, can affect the ability of CaM molecules to access CaMKII subunits [11]. The extension may allow a subunit to sample a volume that is both larger and further away from other subunits, increasing the possibility of a reaction and subsequently leading to more phosphorylated subunits. We did not implement volume exclusion for CaMKII holoenzymes. It is possible that volume exclusion could make the effects of holoenzyme size more apparent.

### Modeling considerations

Our model does not contain Thr305/Thr306 phosphorylation (few subunits would have become phosphorylated there during the time period simulated) and also lacks some newly discovered CaMKII structural feature mechanisms, which may lead to a more complicated activation pattern of CaMKII holoenzyme subunits. For example, it has been found that there exists a compact autoinhibition state, which occurs through dimerization of adjacent subunits from top and bottom rings [53]. Once Ca^2+^-CaM is bound to a dimerized subunit, the dimer disassembles and the two subunits swing away from the center of the holoenzyme. Another recent study indicated that phosphorylated CaMKII subunits can undergo subunit exchange to facilitate propagating activation triggered by Ca^2+^-CaM [54]. The significance of these additional features of CaMKII activation awaits future study. In addition we have not included other Ca^2+^ buffers or CaM-binding proteins in our models (e.g., calbindin, calcineurin, neurogranin, F-actin). Including additional reactions involving these molecules could affect Ca^2+^ concentration, CaM availability and effective Ca^2+^ diffusion, factors that we already analyze separately with the current simulations (Figs 8–12).

One technical challenge for particle-based simulation is to handle diffusion-limited reactions, especially in the presence of highly concentrated molecules. One recent experimental study [26] estimated that the N sites of CaM act very fast to bind Ca^2+^, much faster than previously cited for CaM-N lobe binding kinetics in experimental or modeling studies (although see [55] for a critique of these estimates). If accurate, these fast binding kinetics would place these reactions in the diffusion-limited regime, rendering traditional mass action based methods inaccurate. However, for kinetics this fast, adequate simulation options are limited and not efficient. If using the original Smoldyn algorithm, the simulation time step would have to be considerably reduced to obtain the correct steady state [56]; alternatively, one might increase the geminate recombination probability [37]. Another software package using an enhanced Green’s Function Algorithm [57] can handle the high concentration diffusion-limited reactions accurately, but it takes an impractically long time to run a simulation. If diffusion is slowed considerably in local nanodomains such that Ca^2+^-CaM-CaMKII interactions become diffusion-limited, it will be necessary to develop different algorithms with improved efficiencies to handle these interactions accurately.

## Methods

### Simulator modifications

The modification is based on Smoldyn (V2.37). The reaction and diffusion kinetics of Smoldyn have been thoroughly validated in the past [37]. Since our modification has progressed independently, an additional check is necessary regarding compatibility or integration with updated versions of Smoldyn [38] and its future development.

We expanded the molecule data structure in Smoldyn to include complexes, molecules and binding sites. A complex may contain multiple molecules and a molecule may contain multiple binding sites. Reactions are specified between binding sites. Each binding site has binary states. For example, bound is coded as 1 and unbound as 0; phosphorylated as 1 and unphosphorylated as 0. Each molecule has a vector to store the states of binding sites. All reactions are stored in a hash table with reactants and their binding states as entry keys. A hash table is a data structure that stores association arrays and allows rapid lookup. In our case, the reaction network can be considered as associations between reactants, and therefore is ideal to be implemented using a hash table. CaM is an example of a molecule with multiple binding sites. The binding reactions involving the N and C lobes of CaM can be coded as in Fig 1B. A CaMKII holoenzyme is an example of a complex composed of two 6-subunit rings. Each subunit is a molecule containing binding sites for CaM and phosphorylation. Each ring has a radius of 8 nm and is separated from its direct neighbors at a fixed distance of 8 nm (estimated from [47]). For simplicity, we usually modeled CaMKII holoenzymes as one ring of 6 subunits. A link to the sample reaction configuration files to be run with the modified Smoldyn is included in S1 File. A link to the modified Smoldyn source code is provided in S4 File.

In the original version of Smoldyn, each reaction generates new molecules and reactant molecules are removed. In our case, since one molecule can have multiple binding sites and is potentially associated with multiple partners, entirely removing a molecule is not practical because other attached molecules would also be affected. In addition, removing and generating new molecules makes it difficult to track the reaction history of a molecule. Therefore, during reactions we do not remove molecules but merely change molecule binding states and positions. Molecules bound together physically overlap, synchronize their locations automatically and diffuse together. The diffusion coefficient is determined by the larger molecule. For example, when Ca^2+^ and CaM are bound, the attached Ca^2+^ molecule diffuses with the CaM diffusion rate; similarly, CaM bound to a CaMKII subunit will diffuse with the CaMKII holoenzyme.

Macromolecules usually have multiple binding sites, and sometimes these sites compete for the same ligand. For example, CaM has 4 Ca^2+^ binding sites. Since the N and C sites act independently, the N1 and C1 sites compete for Ca^2+^ and an apoCaM N0C0 can become either N1C0 or N0C1, resulting in a branching reaction scheme. Thus a decision process is needed to choose a reaction path when such a binding event occurs.

To do this, consider the following two reactions

rxn1: camN0C0 + ca <-> camN1C0
rxn2: camN0C0 + ca <-> camN0C1

Rxn1 has a forward rate constant *k*_*f*1_ in μm^−1^ s^−1^ and a backward rate constant *k*_*b*1_ in s^−1^. Rxn2 has similar rate constants *k*_*f*2_ and *k*_*b*2_. The two reactions can be viewed together as an equivalent rxn3, which has overall kinetic rates *k*_*f*3_ and *k*_*b*3_. According to the law of mass action, the reactions can be written as differential equations and we can obtain

$$k_{f3}=k_{f1}+k_{f2}$$ (2)

Smoldyn uses binding radii to implement second order reactions. If two molecules are spatially separated by a distance smaller than the corresponding binding radius, then the reaction proceeds. In Smoldyn, a special algorithm is used to calculate the binding radius, which depends on the kinetic rate constant, simulation time step and total diffusion rate of reactants. In the case of a branched binding scheme sharing common reactants, we first calculate a binding radius *r*_3_ based on *k*_*f*3_. If the distance between a molecule pair is smaller than *r*_3_, binding happens. To make a reaction choice, we generate a uniformly distributed random number from 0 to 1. If the number falls in the range $\left(0,\frac{kf_{1}}{kf_{3}}\right]$, then rxn1 is chosen; instead if the number falls in the range $\left(\frac{kf_{1}}{kf_{3}},1\right]$, we pick rxn2. Following this approach, the network can be kept consistent with the prediction by the mass action law.

### Reaction network

We focus on the interactions among Ca^2+^, CaM, and CaMKII. We first used a Ca^2+^-CaM network for testing to confirm that modifications to the simulator were working properly. Then we added CaMKII holoenzymes to study the reaction network in detail. We set up a box-shaped model to represent a portion of a cell body. The box has dimensions of 1 μm in width and length, as well as 2 μm in depth. The top surface of the box represents the cell membrane, reflective to all molecules. The four sides are also reflective (effectively not different from using periodic boundaries S1 Fig). The bottom surface is partially absorbing to Ca^2+^ ions but reflective to CaM and CaMKII. This conservation of CaM molecules and CaMKII subunits guarantees a steady state initial condition. This partial absorption is a built-in feature in the original Smoldyn to resemble unbounded diffusion [58].

Voltage-gated Ca^2+^ channels (presumably L-type) are located on the top surface to provide Ca^2+^ influx. For simplicity, these channels are placed together at the center of the membrane. The channels open and close depending on a time-varying membrane voltage file generated from a NEURON model (described below). CaMKII subunits are uniformly present at a concentration of 10 μm. They are also immobilized, presumably attached to actin [10]. Freely diffusible CaM molecules are uniformly distributed at a concentration of 5 μm. This is consistent with the notion that at the resting level, freely diffusible CaM molecules are considerably limited in number compared to their binding proteins [48, 50, 59]. S1 Table lists all the reactions with corresponding kinetic parameters involved in the network. Kinetic parameters are integrated from various sources as noted in S1 Table and are adjusted to satisfy microscopic reversibility.

### Ca^2+^ input conditions

**Fig 15:**
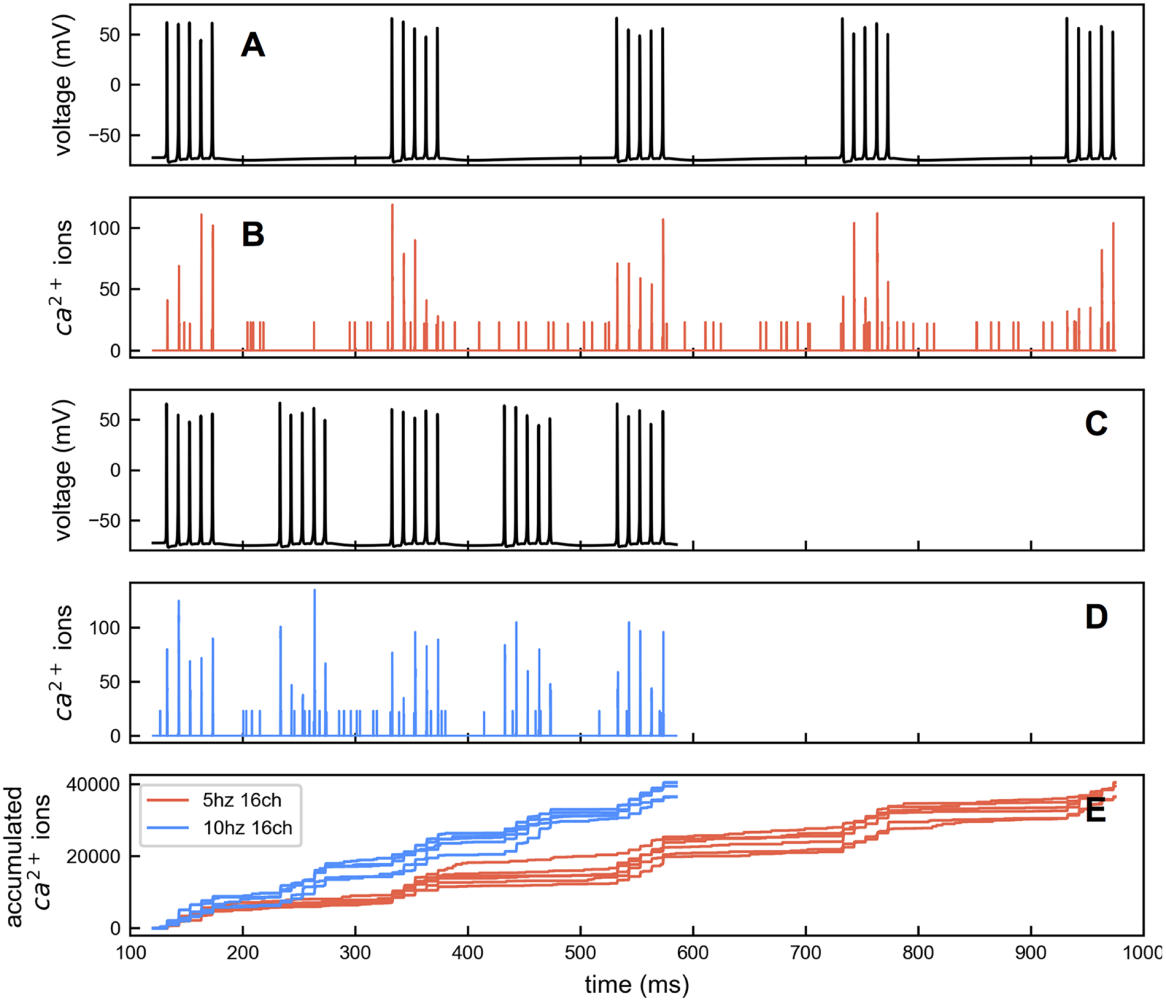
Patterns of membrane voltage and Ca^2+^ influx. (A) A 5 Hz action potential burst voltage file generated from the NEURON model. (B) Ca^2+^ influx generated using the 5 Hz voltage file with 16 Ca^2+^ channels. Ca^2+^ influx is stochastic. There are spontaneous influxes in the absence of action potentials. (C) 10 Hz action potential burst voltage file generated from the NEURON model. (D) Ca^2+^ influx generated using the 10 Hz voltage file with 16 Ca^2+^ channels. (E) Accumulated Ca^2+^ influx for 5 Hz and 10 Hz input with 16 channels. The total Ca^2+^ amount is matched between Ca^2+^ input files.

A model was constructed using NEURON [60] with a detailed morphology of a CA1 pyramidal cell and ion channel conductances to generate a voltage response at the soma to various stimulation conditions. Theta-burst stimulation (5 pulses at 100 Hz, repeated 5 times at 5 Hz or 10 Hz intervals) was applied to synapses on spines taking into account a probability of release measured in experiments [61]. This stimulation activated AMPA and NMDA receptor-channels on dendritic spines causing depolarization in the dendritic tree, which propagated to the soma and initiated action potentials. Soma voltage profiles for 5 Hz and 10 Hz interval stimulation are shown in Fig 15A and 15C.

This membrane voltage output from the NEURON model was used to determine Ca^2+^ influx through L-type Ca^2+^ channels in our reaction network model. Since the kinetics of L-type Ca^2+^ channels are relatively fast and their density is low, the membrane potential is little affected by their activity. Thus the NEURON model and the reaction network model can be safely decoupled. L-type Ca^2+^ channels are modeled stochastically. They open and close in response to the voltage input.

CaL{gate==0} <-> CaL[gate=1].

**Fig 16:**
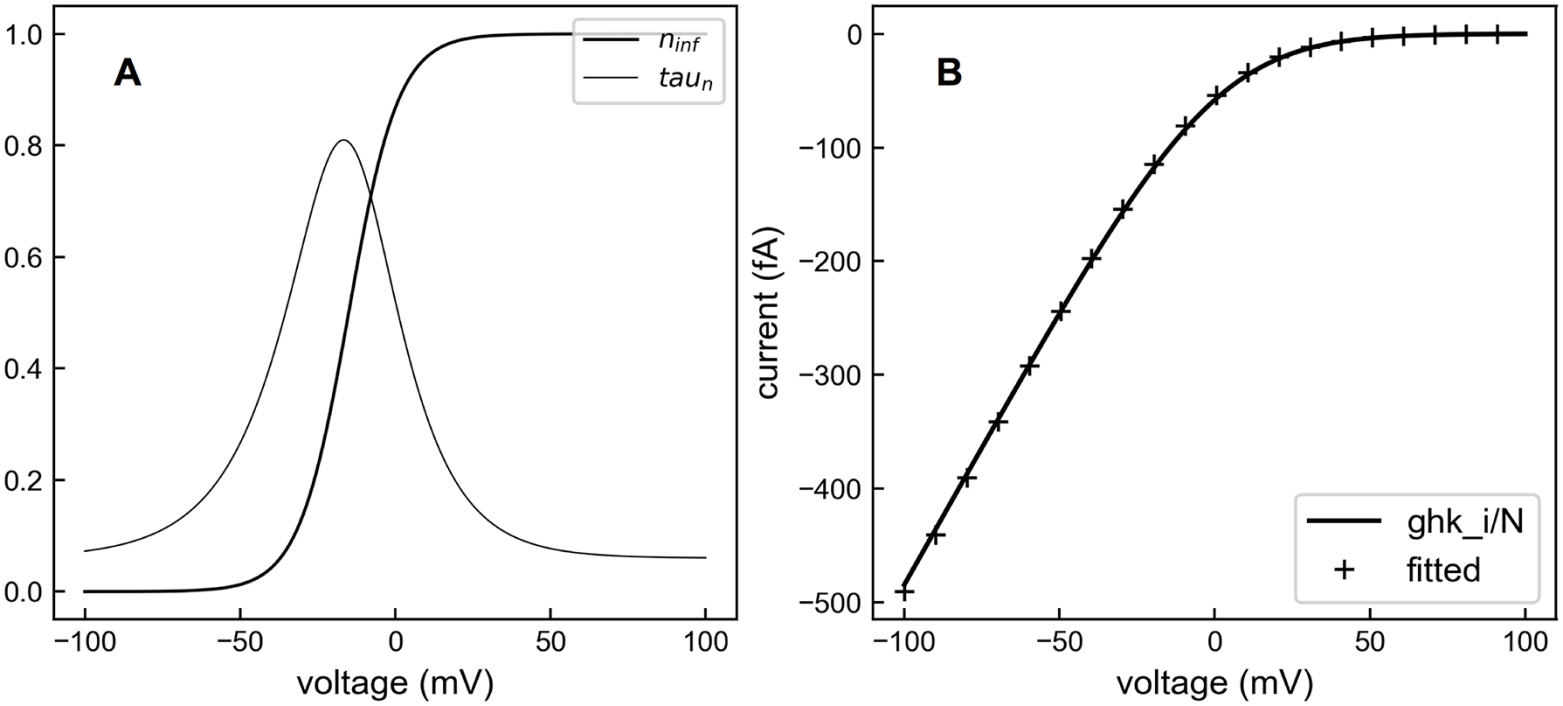
Ca^2+^ channel parameters. (A) *n*_*inf*_(*V*) and *τ*_*n*_(*V*) used to simulate the voltage-gated Ca^2+^ channels. (B) The number of channels N was determined by fitting the single channel current to the Goldman—Hodgkin—Katz (GHK) current equation. N was found to be 16.

The voltage-dependent opening and closing of these channels are modeled with the Hodgkin-Huxley formalism [62]. The rates of channel opening and closing are functions of membrane voltage and are calculated using variables *n*_*inf*_ and *τ*_*n*_, where *n*_*inf*_ describes the steady-state voltage-dependent activation and *τ*_*n*_ is the time constant (Fig 16A).

Then the following set of equations are used to describe voltage-gated Ca^2+^ channel kinetics:

$$rate_{open}\left(V\right)=\frac{n_{\text{inf}}}{\tau_{n}}$$ (3)

$$rate_{close}\left(V\right)=\frac{1-n_{\text{inf}}}{\tau_{n}}$$ (4)

$$n_{\text{inf}}=\frac{1}{1+\text{exp}(-\frac{V-V_{1/2}}{slope})}$$ (5)

$$\tau_{n}=0.06+4\times \frac{0.75\times \sqrt{0.45\times 0.55}}{\text{exp}\left(\left(V-V_{1/2}\right)\times 0.55/slope\right)+\text{exp}\left(-\left(V-V_{1/2}\right)\times 0.45/slope\right)}$$ (6)

where *V*_1/2_ and *slope* together describe the channel activation in response to voltage. In our model, *V*_1/2_ equals -15 mV and *slope* equals 8 mV.

The *rate*_*open*_ and *rate*_*close*_ are used to calculate conditional probabilities to determine the state of a channel for the next time step in the following way

$$P\left(C|O\right)=1-\text{exp}(-rate_{close}\left(V\right)dt)$$ (7)

$$P\left(O|C\right)=1-\text{exp}(-rate_{open}\left(V\right)dt)$$ (8)

P(O|O)=1-P(C|O) (9)

For each channel, at a given time, a probability is calculated based on the membrane voltage to decide whether a channel opens. If it opens, a varying number of Ca^2+^ ions enter. To calculate how many ions, we used the following equation [52] to obtain the unitary current for a single channel

$$i_{ca}=-g\left(V-V_{s}\right)\frac{\text{exp}(\frac{-(V-V_{s})}{RT/zF})}{1-\text{exp}(\frac{-(V-V_{s})}{RT/zF})}$$ (10)

where *g* is chosen as 5 pS and *RT*/*zF* equals 12 mV, and *V*_*s*_ is determined as described below. A current density is calculated using Goldman—Hodgkin—Katz current equation as follows,

$$I=P\frac{Vz^{2}F^{2}}{RT}\frac{{\left[Ca^{2+}\right]}_{i}-{\left[Ca^{2+}\right]}_{o}\text{exp}\left(-zVF/RT\right)}{1-\text{exp}(-zVF/RT)}$$ (11)

where extracellular [Ca^2+^]_o_ equals 2 mM, intracellular [Ca^2+^]_i_ equals 50 nm and a maximum membrane permeability to Ca^2+^ *P* is 0.241 × 10^−3^ cm s^−1^. Since the membrane surface is 1 μm^2^, the current density is converted to a total current *I*_*ghk*_ for this area. If a total of N channels are present, in this membrane surface, the single channel current *i*_*ca*_ equals $\frac{I_{ghk}}{N}$. By fitting the total current *I*_*ghk*_ with *N* × *i*_*ca*_, we obtained an N of 16 and a *V*_*s*_ of −1.91 mV (Fig 16B). From *i*_*ca*_, the number of ions entering each open channel during one time step is calculated as $\frac{i_{ca}}{2e}dt$ (Fig 15B and 15D), where 2 is the valence of Ca^2+^ and *e* is the elementary charge. To guarantee a consistent amount of total Ca^2+^ for a given set of 5 Hz and 10 Hz voltage files, we generated 40 trials of Ca^2+^ influx files for each frequency and then selected the ones with equivalent total Ca^2+^ influx (Fig 15E).

## Supporting information

S1 File Descriptions of all models used in the study. (PDF)

S2 File How the combinatorics numbers are calculated. (PDF)

S3 File Neighbor sensitivity of CaMKII autophosphorylation. (PDF)

S4 File Source code of the modified Smoldyn. Please follow the GitHub link (https://github.com/lxm1117/smoldyn-cplx) to the source code repository for future updates. More links to the relevant online repositories are in S1 File.
(GZ) Click here for additional data file.

S1 Fig Comparison of CaMKII subunits phosphorylated and CaM bound simulated with reflective and periodic side surfaces. We see that because geometry is a box, reflective and periodic boundary conditions give comparable results. (A) Phosphorylated CaMKII subunits from 10 trials with reflective boundary conditions and periodic boundary conditions (B) The same as in A but for CaM-bound CaMKII subunits.
(TIFF)

S2 Fig The modified Smoldyn produces results consistent with the formula [22]. Holoenzymes has 2, 3, 6, or 12 subunits. Fully loaded CaM saturated CaMKII. The percentage of phosphorylated subunits are plotted over time.
(TIFF)

S3 Fig The modified Smoldyn produces results consistent with those computed with a spatial Gillespie algorithm. (A) Bound CaMKII subunits simulated separately using the spatial Gillespie algorithm (red line) and modified Smoldyn (blue line) (B) The same as in A but for phosphorylated CaMKII subunits.
(TIFF)

S4 Fig Probability of release at synapses used in the NEURON model. (A) Probability of release measured experimentally [61] using high frequency stimulation (100 Hz) as a function of stimulation pulse number. (B) Probability of release profile used to generate theta-burst stimulation at various frequencies. Long interval can be adjusted to give rise to 5 Hz or 10 Hz action potential patterns.
(TIFF)

S5 Fig Morphology of the pyramidal neuron used in the NEURON model. Soma is colored in blue and dendritic spines are marked in black. Probabilistic synaptic input as in S4 Fig is given at spines. Action potentials are recorded in the soma and used to generate Ca^2+^ influx.
(TIFF)

S6 Fig Accumulated reaction occurrences generated from 6 trials for CaM CaMKII binding reactions in the presence of 5 Hz and 10 Hz Ca^2+^ input. This is a rearrangement of the same data shown in Fig 7A and 7B.
(TIFF)

S1 Table Kinetic parameters of all reactions. (PDF)
