# Supplementary material for: Biophysical attributes that affect CaMKII activation deduced with a novel spatial stochastic simulation approach
Source: PLoS Comput Biol. 2018 Feb 5;14(2):e1005946. doi: 10.1371/journal.pcbi.1005946 (PMC5814094; doi:10.1371/journal.pcbi.1005946)
Supplement: S1 File — (PDF) [file pcbi.1005946.s001.pdf]

## Supplement File S1

## Kinetic Parameters and descriptions of all models used in the study

**Online repositories:**

Source code of modified Smoldyn

Configurations files to run the main model

NEURON model configuration files

Relevant files for the spatial Gillespie model

**Parameters Model 1**

Purpose: to test and compare with COPASI

Geometry: 500 nm x 500 nm x 500 nm

Surface conditions: all sides reflective

How $Ca^{2+}$ ions are added: uniformly distributed in the cube at the initial condition.

Initial molecule concentrations:

$Ca^{2+}$ ion number = 3000

$[Ca^{2+}]$= 39.867 uM

CaM molecule number = 700

[CaM]= 9.302 uM

Molecule diffusions:

$D_{ca}$= 220000 $nm^2$/ms = 2.2 x $10^{-6}$ $cm^2$/s (Allbritton et al., 1992)

slowed $Ca^{2+}$ diffusion condition:

$D_{ca\_slow}$= 110000 $nm^2$/ms = 1.1 x $10^{-6}$ $cm^2$/s

$D_{cam}$= 50000 $nm^2$/ms = 50 $nm^2$/us = 0.5 x $10^{-6}$ $cm^2$/s (Faas et al., 2011; Sanabria et al. 2008 )

Time step:

dt= 0.001 ms for normal $D_{ca}$ condition

dt= 0.0005 ms for $D_{ca\_slow}$ condition

Reactions included:

ca + N0C0 <-> N1C0

ca + N0C0 <-> N0C1

ca + N1C0 <-> N2C0

ca + N1C0 <-> N1C1
ca + N0C1 <-> N1C1
ca + N0C1 <-> N0C2
ca + N2C0 <-> N2C1
ca + N1C1 <-> N1C2
ca + N1C1 <-> N2C2
ca + N0C2 <-> N1C2
ca + N2C1 <-> N2C2
ca + N1C2 <-> N2C2

Binding kinetics are the same as listed in Table S1 at the end of the file. See Fig 1C in the text for testing results. No volume exclusion simulated.

**Parameters Model 2**
Purpose:
The test is to compare results with the theoretical steady state in Michalski and Loew (2012). Assuming that autophosphorylations occurs unidirectionally and only between subunits that are both bound with CaM, then in the long run the fraction of phosphorylated subunits approaches $F_p(N)$ for a holoenzyme with N subunits (eq.8 in Michalski and Loew). As N goes to infinity, the fraction $F_p(N)$ approaches $1-e^{-1}$. We set up a scenario to test the steady state by initializing all CaMKII subunits to be bound with camN2C2.

Geometry: 1 um x 1 um x 1 um
Surface conditions: all sides reflective
How $Ca^{2+}$ ions are added: uniformly distributed in the cube at the initial condition.

Initial molecule concentration:
N2C2 molecule number = 6020
[N2C2] = 10 uM
CaMKII molecule number = 6000
[CaMKII] = 9.9668 uM

Molecule diffusions:
$D_{cam}$ = 50000 nm$^2$/ms
$D_{camkii}$= 0 nm$^2$/ms (immobilized as in the complete model)

Reactions included:
N2C2 + CaMKII <-> KN2C2
$CaMKII_{bound}$ -> $CaMKII_{phos}$, only if the neighbor subunit is $CaMKII_{bound}$

Time step: dt= 0.001 ms

See Fig S2 for results. No volume exclusion simulated.

**Parameters Model 3**
Purpose: to test and compare with a previously published Gillespie simulation (Zeng and Holmes, 2010)
Geometry: 1 um x 1 um x 1 um
Surface conditions: all sides reflective

How $Ca^{2+}$ ions are added:
uniformly distributed in the cube at the initial condition

Initial molecule concentration:
camN0C0 molecule number = 3000
[camN0C0] = 4.9834 uM
CaMKII molecule number = 6000
[CaMKII] = 9.9668 uM
$Ca^{2+}$ ion number = 6000
$[Ca^{2+}]$ = 9.9668 uM

Molecule diffusions:
$D_{ca}$ = 220000 $nm^2$/ms
$D_{cam}$ = 50000 $nm^2$/ms
$D_{camkii}$= 0 $nm^2$/ms (immobilized as in the complete model)

Reactions included:
the set of reactions as in the complete model

Time step:
dt= 0.001 ms for normal Dca condition

See Fig S3 for results.
No volume exclusion simulated.
The spatial Gillespie model used the same parameters and geometry as above except for the time step which is not relevant for this code. A version of the code for a 6 subunit CaMKII holoenzyme is available on GitHub.

**Parameters Model 4 (Main model)**
Purpose: to study how $Ca^{2+}$ affects CaMKII activation pattern
Geometry: 1 um x 1 um x 2 um

Surface conditions:
The top side serves as the membrane surface, where $Ca^{2+}$ channels are located. The four side surfaces are reflective (not significantly different from using periodic boundary, Fig S1). The bottom surface is reflective to CaM and CaMKII, but unbounded diffusion for $Ca^{2+}$ ions. Conservation of CaM and CaMKII allows the system to start at a steady state initial condition.

How $Ca^{2+}$ ions are added:
$Ca^{2+}$ ions flow in from the top membrane surface as a single source, either in 5 Hz or 10 Hz patterns

Initial molecule concentration:
N0C0 molecule number = 5825
[N0C0] = 5.004 uM
CaMKII subunits number = 11841 (grouped in 6 subunits)
[CaMKII]monomer = 9.835 uM
KN0C0 molecule number = 195
[KN0C0]= 0.162 uM

Molecule diffusions:
$D_{ca}$ = 220000 $nm^2$/ms
$D_{cam}$ = 50000 $nm^2$/ms
$D_{camkii}$= 0 $nm^2$/ms (immobilized as in the complete model)

Reactions included:
Described as in Table S1.
No volume exclusion simulated.

**Parameters Model 5 (NEURON model)**

The NEURON model was built previously to study how synaptic stimulation patterns affect somatic $Ca^{2+}$ influx. The model uses a detailed pyramidal cell morphology (Fig S5). There are 190 spine synapses in total. Each spine has 200 AMPARs and 10 NMDARs. In the present study, we use this model to generate soma action potentials with four 100 Hz pulses delivered at intervals of 5 Hz and 10 Hz. Synaptic release is probabilistic and follows an experimentally measured probability sequence (Fig S4A). We use the first four probabilities and adapted them for Theta-burst stimulations (Fig S4B).

The relevant files are available on GitHub

**Parameters Model 6**

Description: a reduced network model tested using Smoldyn to confirm the effect of saturating $Ca^{2+}$.

Geometry: 1 um x 1 um x 2 um

Surface conditions:

The top side serves as the membrane surface, where $Ca^{2+}$ channels are located. The four side surfaces are reflective. The bottom surface is reflective to CaM and CaMKII but unbounded diffusion for $Ca^{2+}$ ions. Conservation of CaM and CaMKII allows the system to start at a steady state initial condition.

How $Ca^{2+}$ ions are added:

$Ca^{2+}$ ions flow in from the top membrane surface as a single source, either in 5 Hz or 10 Hz patterns.

Initial molecule concentration:

camN0C0 molecule number = 6020

[camN0C0] = 5 uM

CaMKII subunits number = 12036

[CaMKII] = 9.997 uM

Molecule diffusions:

$D_{ca}$ = 220000 $nm^2$/ms

$D_{cam}$ = 50000 $nm^2$/ms

$D_{camkii}$= 0 $nm^2$/ms (immobilized as in the complete model)

Time step: dt= 0.001 ms

Reactions included:
A reduced network as in Fig 13. No volume exclusion simulated.
