# Supplementary material for: Biophysical attributes that affect CaMKII activation deduced with a novel spatial stochastic simulation approach
Source: PLoS Comput Biol. 2018 Feb 5;14(2):e1005946. doi: 10.1371/journal.pcbi.1005946 (PMC5814094; doi:10.1371/journal.pcbi.1005946)
Supplement: S2 File — (PDF) [file pcbi.1005946.s002.pdf]

File S2

Combinatorial explosion and the necklace function

A problem of combinatorial explosion arises when reactants belong to multi-subunit multi-state macromolecules. An example is CaMKII holoenzymes. Each holoenzyme consists of 2 rings of 6 subunits. When interacting with CaM, there are two types of activities that can occur for each subunit: CaM binding and phosphorylation. This leads to four distinct states per subunit. However, CaM binding to CaMKII also depends on Ca^{2+} binding to CaM and this gives rise to more distinct states. Assuming the N and C lobes of CaM bind Ca^{2+} independently and Ca^{2+} binding within a lobe is cooperative [1], we get 9 distinct binding states of CaM. Also, different CaM binding states have distinct binding and phosphorylation kinetics with CaMKII subunits. Taken together this means that each subunit can be bound with CaM in one of the 9 configurations, or phosphorylated and bound with CaM in one of 9 configurations, or unbound and phosphorylated, or unbound and unphosphorylated, giving 20 possible states. This level of kinetic detail per subunit adds to the total possible unique states of the CaMKII holoenzyme, and the total number of states increases rapidly as the number of subunits in the holoenzyme grows.

The total possible states can be calculated with the Necklace number formula used in combinatorics as follows

$$N(a,n) = \frac{1}{n}\sum_{i=1}^{v(n)} \phi(d_i) a^{n/d_i} \tag{1}$$

where n is the number of subunits on the ring (or beads on a necklace), a is the number of possible states each subunit has and d_i are the divisors of n. $\phi(d_i)$ is the Euler totient function. It calculates the number of positive integers that are less than d_i and prime to d_i.

In our case, we model only one ring of the CaMKII holoenzyme, and therefore $n = 6$. Starting with a simple situation, suppose each subunit has only 4 states: no CaM bound and unphosphorylated, CaM bound only, CaM bound and phosphorylated, and phosphorylated only. Thus $a = 4$. The divisors of $n = 6$ are 1, 2, 3, and 6; the corresponding totient numbers for each divisor are 1, 1, 2 and 2 respectively. As a result, $N(n = 6, a = 4) = \frac{1}{6}(1 \times 4^{6/1} + 1 \times 4^{6/2} + 2 \times 4^{6/3} + 2 \times 4^{6/6}) = 700$ unique states of the CaMKII holoenzyme. If we consider all of the distinct Ca^{2+}-CaM states, then for each CaMKII subunit we could have 9 CaM bound states, 9 CaM bound and phosphorylated states, 1 unbound and unphosphorylated state, as well as 1 unbound and phosphorylated state. Thus $a = 20$. Correspondingly, $N(n = 6, a = 20) = \frac{1}{6}(1 \times 20^{6/1} + 1 \times 20^{6/2} + 2 \times 20^{6/3} + 2 \times 20^{6/6}) = 10668140$. For an individual subunit, an increase of possible states from 4 to 20 may not seem overwhelming. However, the resulting growth in combinations of holoenzyme states from 700 to 10668140 raises a problem if one tries to describe a network involving multistate marcromolecules.

References

1. Linse S, Helmersson A, Forsén S. Calcium binding to calmodulin and its globular domains. The Journal of biological chemistry. 1991;266(13):8050–8054.
