# Supplementary material for: Biophysical attributes that affect CaMKII activation deduced with a novel spatial stochastic simulation approach
Source: PLoS Comput Biol. 2018 Feb 5;14(2):e1005946. doi: 10.1371/journal.pcbi.1005946 (PMC5814094; doi:10.1371/journal.pcbi.1005946)
Supplement: S3 File — (PDF) [file pcbi.1005946.s003.pdf]

File S3

The neighbor sensitivity of CaMKII autophosphorylation

Software such as BioNetGen can generate reaction networks using a rule-based approach. However, simply expanding the network does not solve the combinatorial problem for CaMKII holoenzymes. The problem for multi-subunit CaMKII arises from its neighbor sensitivity. The concept ”neighbor” is a relative one instead of an absolute one. With the standard rule-based approach, there is no alternative way to describe the idea ”neighbor” than specifying the neighbor for each subunit. The modified Smoldyn allows a subunit to read the binding or phosphorylation state of its neighbor through a pointer that links to those neighbor sites. Therefore the neighbor subunits sites can be ”sensed” directly and the neighbor sensitivity in autophosphorylation can be described without specifying the neighbor subunit. As an example, neighbor sensitivity and autophosphorylation in the main model is written in the following way,

```
camkii{cam==1,P==0,n.P==1,n.cam==0}~cam{K==1,Kp==0} → camkii[P=1]~cam[Kp=1]
camkii{cam==1,P==0,n.P==0,n.cam==1}~cam{K==1,Kp==0} → camkii[P=1]~cam[Kp=1]
camkii{cam==1,P==0,n.P==1,n.cam==1}~cam{K==1,Kp==0} → camkii[P=1]~cam[Kp=1].
```

The reactant conditions for the three reactions can be read respectively as ”an unphosphorylated CaMKII subunit that is CaM bound and its neighbor is phosphorylated but not CaM bound”, ”an unphosphorylated CaMKII subunit that is CaM bound and its neighbor is CaM bound but not phosphorylated”, and ”an unphosphorylated CaMKII subunit that is CaM bound while its neighbor is both phosphorylated and CaM bound”.

The n.P site and n.cam site of CaMKII are user-defined to reflect the binding and phosphorylation status of the neighbor subunit. They are merely functional and not actual binding sites. The Kp site of CaM is also updated in the reaction to track the phosphorylation status of the CaMKII subunit. The functional sites allow one to describe the autophosphorylation of a CaMKII subunit depending on the state of CaM attached to the subunit. Thus phosphorylation rate can be CaM state dependent. For example, it is possible to specify a reaction as ”a CaMKII subunit that has a phosphorylated neighbor and is bound to a CaM in the fully loaded state” using the following expression

```
camkii{cam==1,n.P==1,P==0}~cam{K==1,Kp==0,N1==1,N2==1,C1==1,C2==1}.
```
