# Supplementary figures and images for: Biophysical attributes that affect CaMKII activation deduced with a novel spatial stochastic simulation approach
Source: PLoS Comput Biol. 2018 Feb 5;14(2):e1005946. doi: 10.1371/journal.pcbi.1005946 (PMC5814094; doi:10.1371/journal.pcbi.1005946)

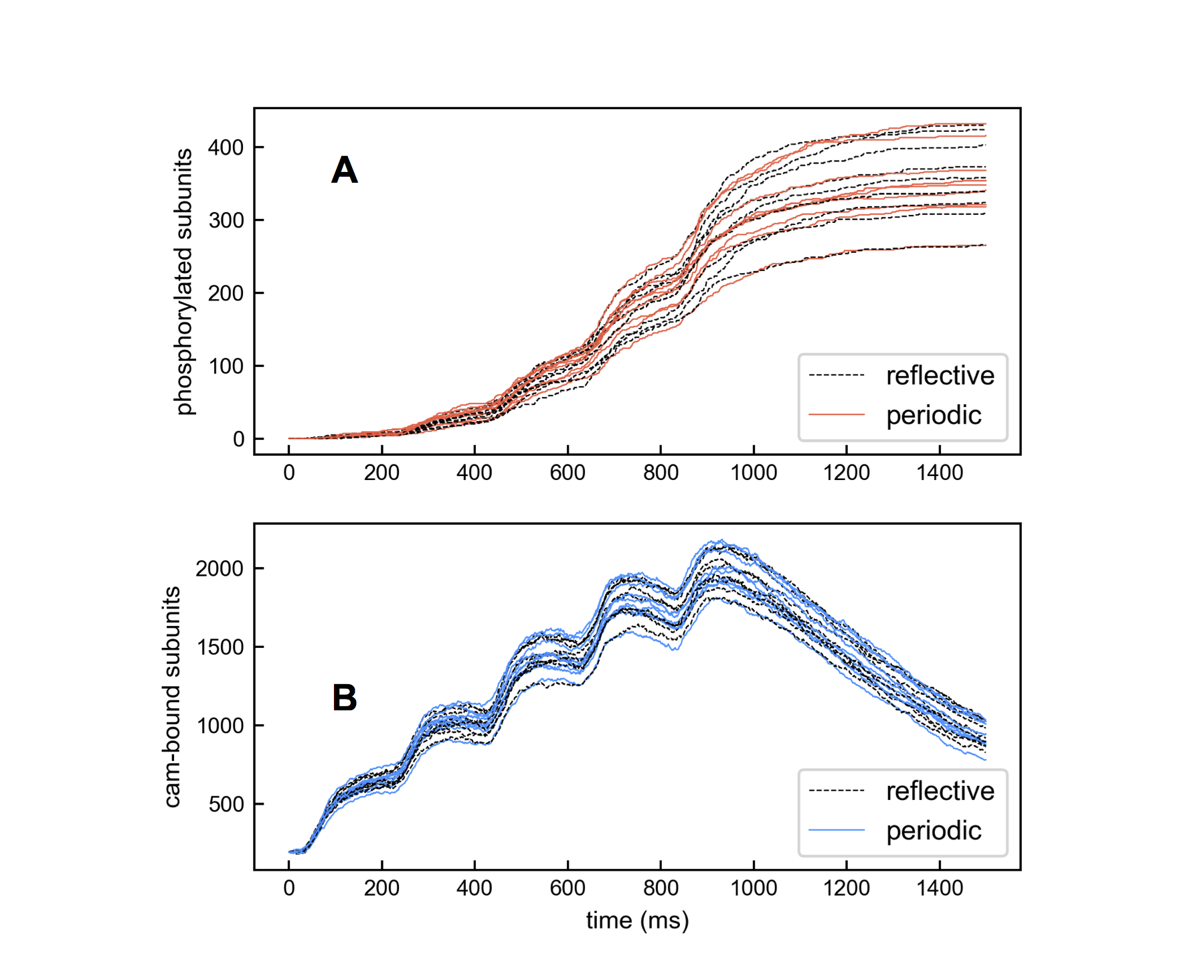

Supplement: S1 Fig — We see that because geometry is a box, reflective and periodic boundary conditions give comparable results. (A) Phosphorylated CaMKII subunits from 10 trials with reflective boundary conditions and periodic boundary conditions (B) The same as in A but for CaM-bound CaMKII subunits. (TIFF) [file pcbi.1005946.s005.tiff]

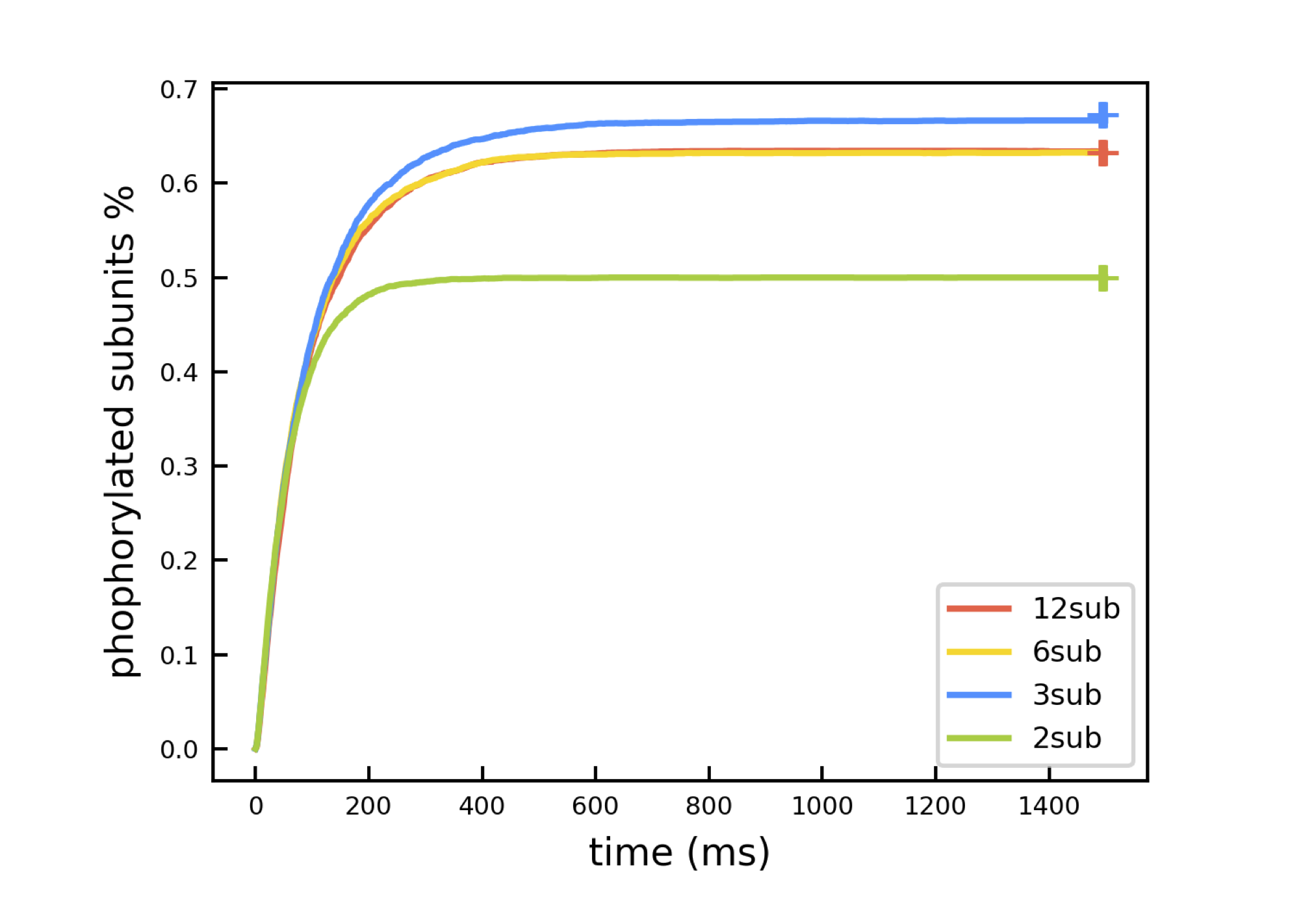

Supplement: S2 Fig — Holoenzymes has 2, 3, 6, or 12 subunits. Fully loaded CaM saturated CaMKII. The percentage of phosphorylated subunits are plotted over time. (TIFF) [file pcbi.1005946.s006.tiff]

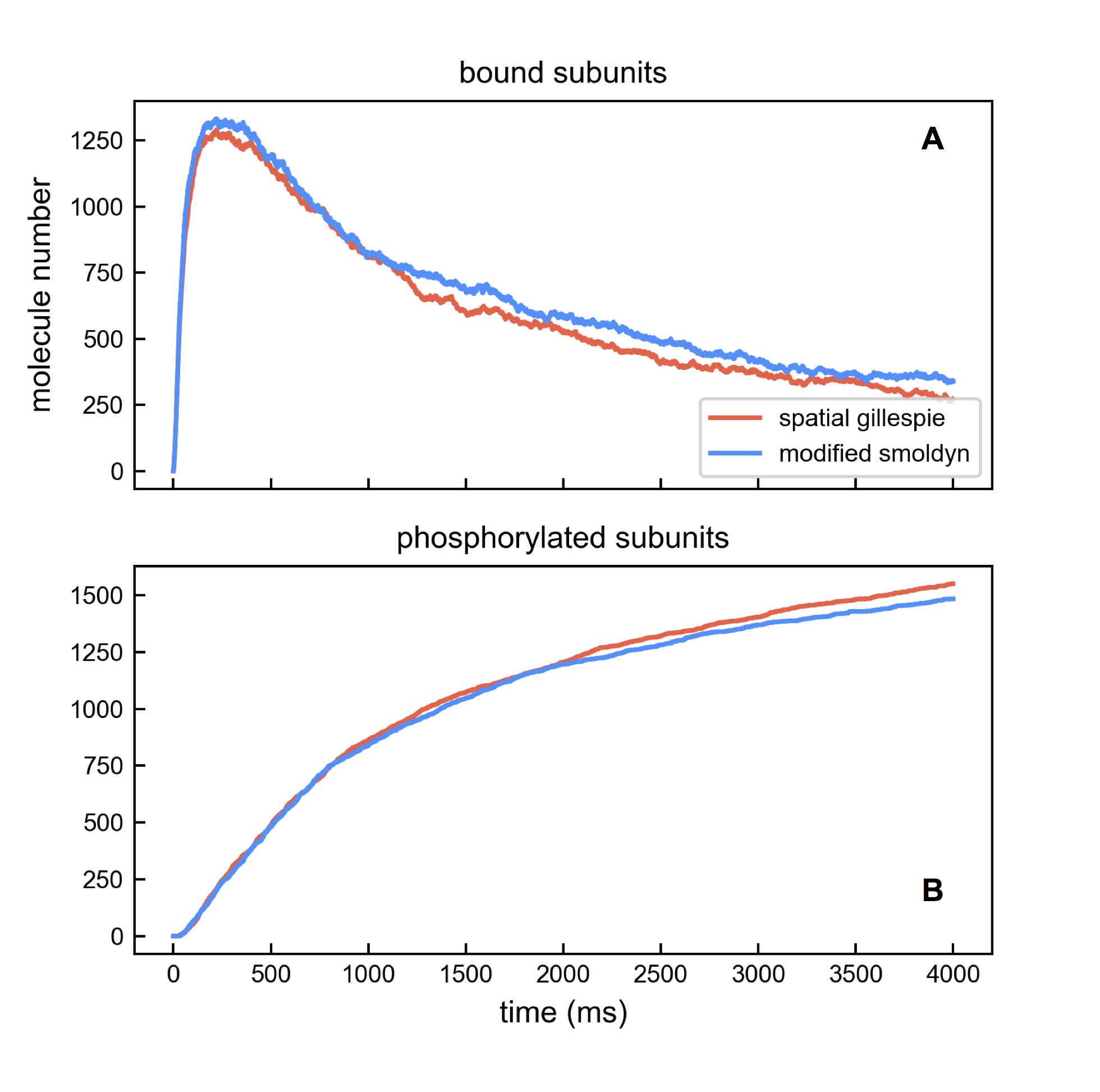

Supplement: S3 Fig — (A) Bound CaMKII subunits simulated separately using the spatial Gillespie algorithm (red line) and modified Smoldyn (blue line) (B) The same as in A but for phosphorylated CaMKII subunits. (TIFF) [file pcbi.1005946.s007.tiff]

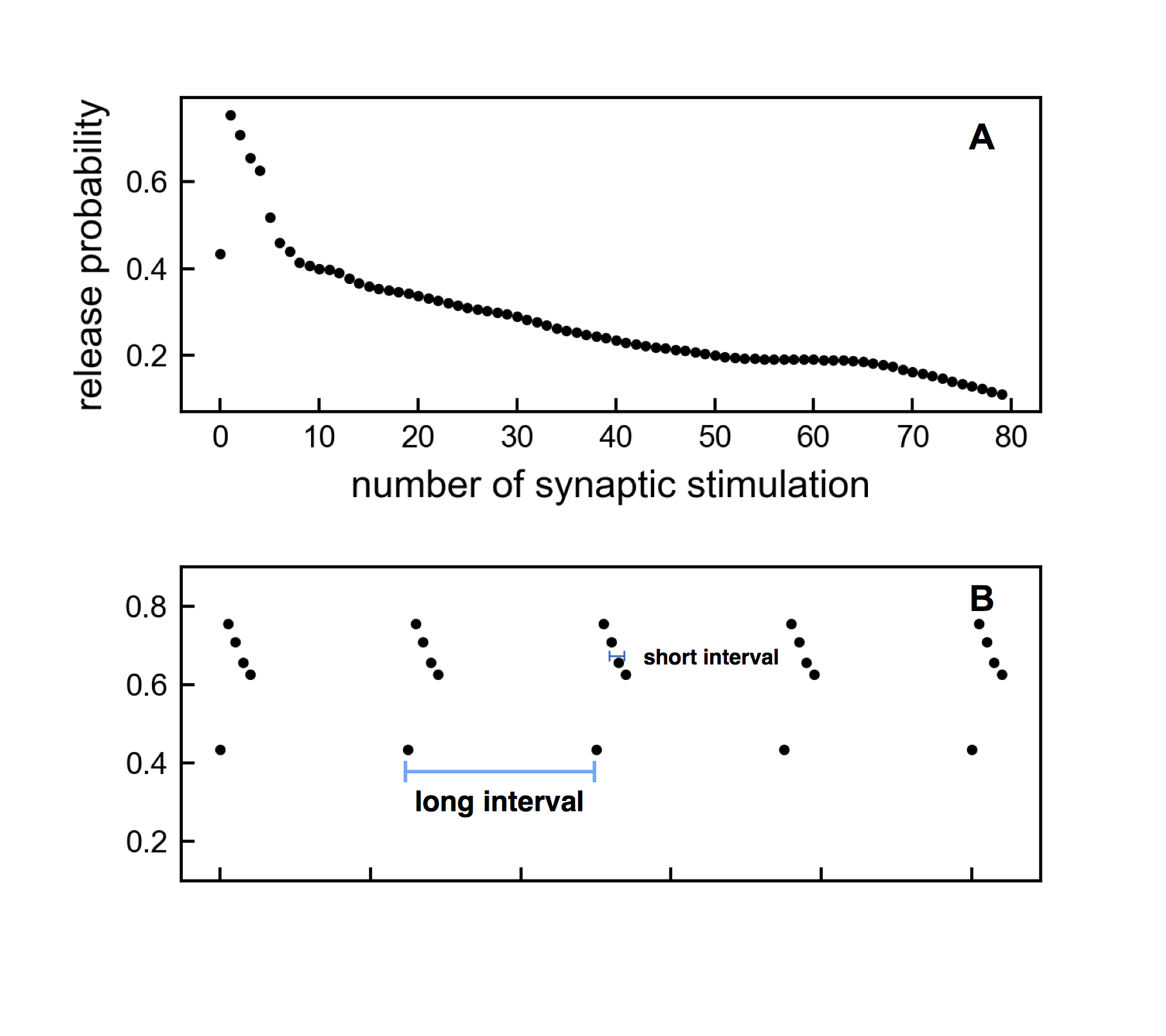

Supplement: S4 Fig — (A) Probability of release measured experimentally [61] using high frequency stimulation (100 Hz) as a function of stimulation pulse number. (B) Probability of release profile used to generate theta-burst stimulation at various frequencies. Long interval can be adjusted to give rise to 5 Hz or 10 Hz action potential patterns. (TIFF) [file pcbi.1005946.s008.tiff]

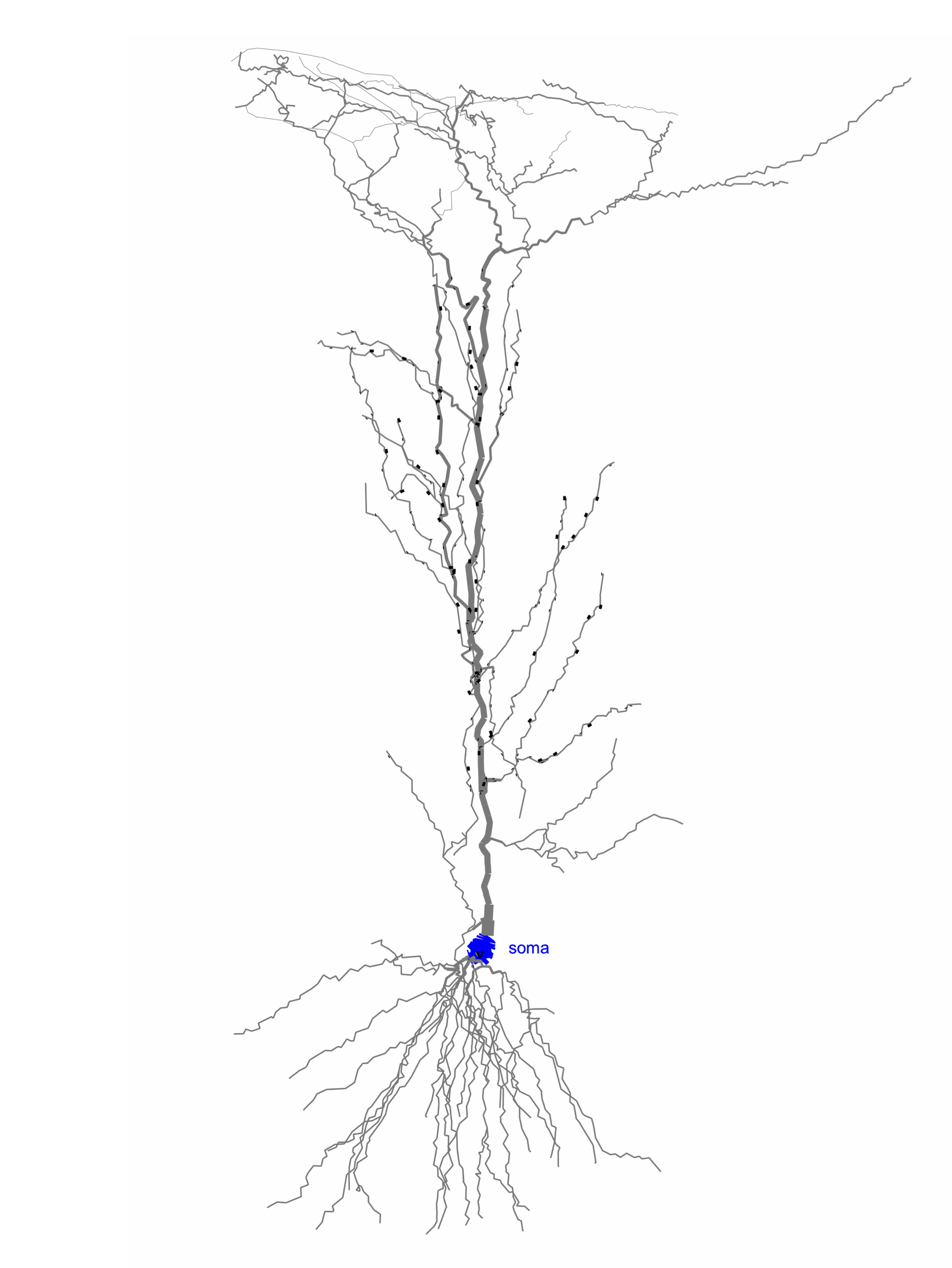

Supplement: S5 Fig — Soma is colored in blue and dendritic spines are marked in black. Probabilistic synaptic input as in S4 Fig is given at spines. Action potentials are recorded in the soma and used to generate Ca2+ influx. (TIFF) [file pcbi.1005946.s009.tiff]

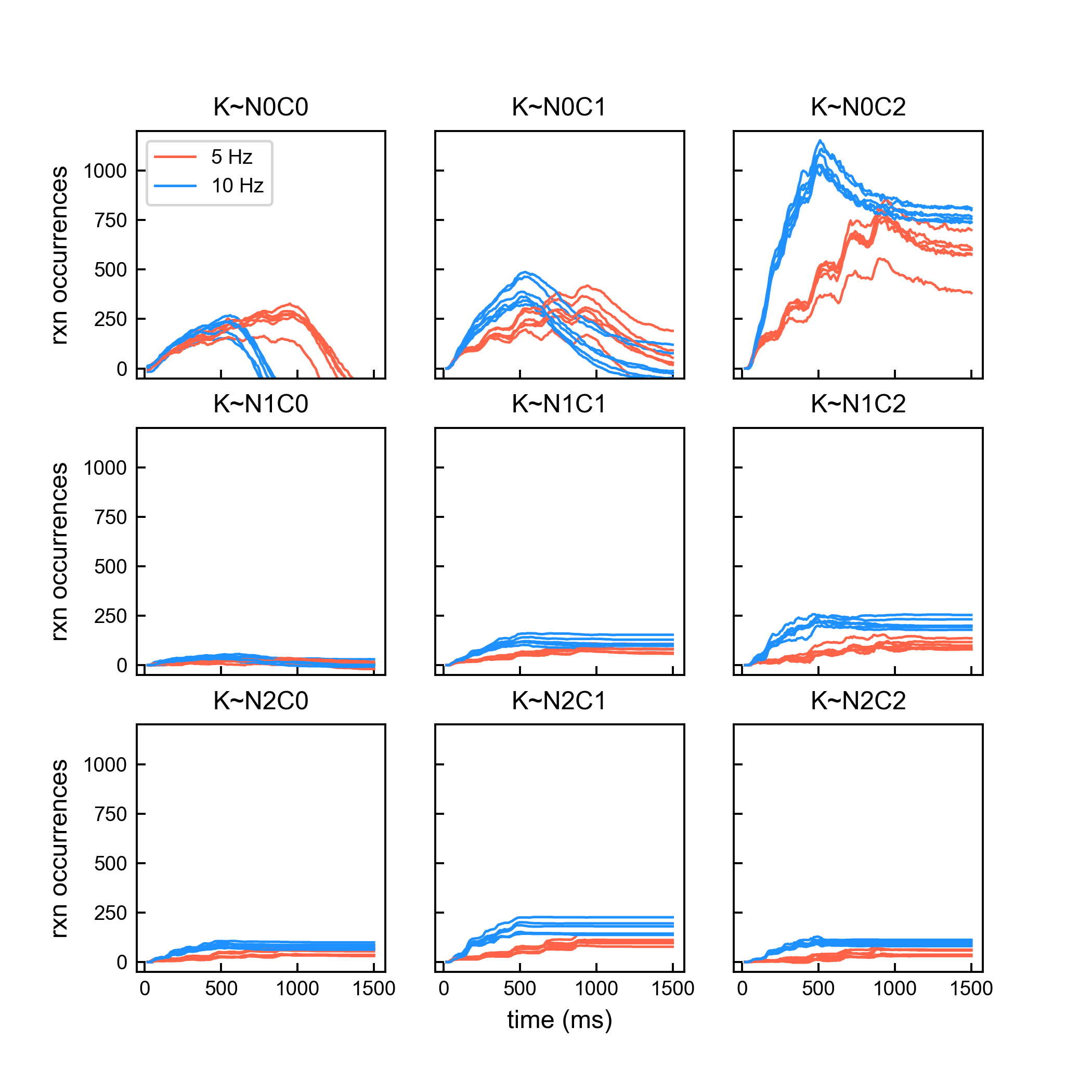

Supplement: S6 Fig — This is a rearrangement of the same data shown in Fig 7A and 7B. (TIFF) [file pcbi.1005946.s010.tiff]
