# Supplementary material for: Biophysical attributes that affect CaMKII activation deduced with a novel spatial stochastic simulation approach
Source: PLoS Comput Biol. 2018 Feb 5;14(2):e1005946. doi: 10.1371/journal.pcbi.1005946 (PMC5814094; doi:10.1371/journal.pcbi.1005946)
Supplement: S1 Table — (PDF) [file pcbi.1005946.s011.pdf]

Table S1. Kinetic parameters of all reactions

	on			off			kD
reaction name	nm^3ms^{-1}	$uM^{-1}s^{-1}$	$uM^{-1}s^{-1}$	ms^{-1}	s^{-1}	s^{-1}	uM
N1~ca	161500	95	adapted [21], [20]	3.75	3,750.00	adapted [21], [20]	39.47368
N2~ca	161500	95	adapted [21], [20]	0.75	750	adapted [21], [20]	7.89474
C1~ca	8548.5714	5.02857	adapted [21], [20]	0.0528	52.8	adapted [21], [20]	10.50000
C2~ca	8548.5714	5.02857	adapted [21], [20]	0.00784	7.84	adapted [21], [20]	1.55909
KN1~ca	129200	***76***	[21]	0.3	***300***	[21]	3.94737
KN2~ca	129200	***76***	[21]	0.06	***60***	[21]	0.78947
KC1~ca	74800	***44***	[21]	0.033	***33***	[21]	0.75000
KC2~ca	74800	***44***	[21]	0.0049	***4.9***	[21]	0.11136
KpN1~ca	129200	76	adapted [21], [20]	0.06	60	microscopic reversibility	0.78947
KpN2~ca	129200	76	adapted [21], [20]	0.03	30	microscopic reversibility	0.39474
KpC1~ca	74800	44	adapted [21], [20]	0.0066	6.6	microscopic reversibility	0.15000
KpC2~ca	74800	44	adapted [21], [20]	0.00245	2.45	microscopic reversibility	0.05568
K~N0C0	32.3	0.019	[21]	0.005586	***5.586***	[21]	294
K~N0C1	501.5	0.295	[21]	0.006195	***6.195***	[21]	21
K~N0C2	7820	4.6	[21]	0.0069	***6.9***	[21]	1.5
K~N1C0	187	0.11	[21]	0.003234	***3.234***	[21]	29.4
K~N1C1	2805	1.65	[21]	0.003465	***3.465***	[21]	2.1
K~N1C2	44200	26	[21]	0.0039	***3.9***	[21]	0.15
K~N2C0	1020	0.6	[21]	0.001764	***1.764***	[21]	2.94
K~N2C1	16150	9.5	[21]	0.001995	***1.995***	[21]	0.21
K~N2C2	131750	77.5	[21]	0.0011625	***1.1625***	[21]	0.015
Kp~N0C0	32.3	0.019	adapted [21], [20]	0.00016758	0.16758	microscopic reversibility	8.82
Kp~N0C1	501.5	0.295	adapted [21], [20]	0.00003717	0.03717	microscopic reversibility	0.126
Kp~N0C2	7820	4.6	adapted [21], [20]	0.0000207	0.0207	microscopic reversibility	0.0045
Kp~N1C0	187	0.11	adapted [21], [20]	0.000019404	0.019404	microscopic reversibility	0.1764
Kp~N1C1	2805	1.65	adapted [21], [20]	0.000004158	0.004158	microscopic reversibility	0.00252
Kp~N1C2	44200	26	adapted [21], [20]	0.00000234	0.00234	microscopic reversibility	0.00009
Kp~N2C0	1020	0.6	adapted [21], [20]	0.000005292	0.005292	microscopic reversibility	0.00882
Kp~N2C1	16150	9.5	adapted [21], [20]	0.000001197	0.001197	microscopic reversibility	0.000126
Kp~N2C2	131750	77.5	adapted [21], [20]	0.00000034875	0.00034875	microscopic reversibility	0.0000045
KN0C0~p				0	0	[21]	
KN0C1~p				0.0005	0.5	microscopic reversibility	
KN0C2~p				0.001	1	microscopic reversibility	
KN1C0~p				0.0005	0.5	microscopic reversibility	
KN1C1~p				0.0025	2.5	microscopic reversibility	
KN1C2~p				0.005	5	microscopic reversibility	
KN2C0~p				0.001	1	microscopic reversibility	
KN2C1~p				0.005	5	adapted [21], [20]	
KN2C2~p				0.01	10	adapted [21], [20], [19], [12]	
KpNxCy_dephos				0.000003	0.003	[20]	
Kp_dephos				0.000003	0.003	[20]	

Remarks: The ***bold italic*** parameters are directly from ref [21]. The remaining parameters are either derived or adapted from both references or constrained with microscopic reversibility.
